# Endogenous Human MDM2-C Is Highly Expressed in Human Cancers and Functions as a p53-Independent Growth Activator

**DOI:** 10.1371/journal.pone.0077643

**Published:** 2013-10-11

**Authors:** Danielle R. Okoro, Nicoleta Arva, Chong Gao, Alla Polotskaia, Cindy Puente, Melissa Rosso, Jill Bargonetti

**Affiliations:** Department of Biological Sciences Hunter College and The Graduate Center Departments of Biology and Biochemistry, City University of New York, New York City, United States of America; Virginia Commonwealth University, United States of America

## Abstract

Human cancers over-expressing *mdm2*, through a T to G variation at a single nucleotide polymorphism at position 309 (*mdm2* SNP309), have functionally inactivated p53 that is not effectively degraded. They also have high expression of the alternatively spliced transcript, *mdm2-C*. Alternatively spliced *mdm2* transcripts are expressed in many forms of human cancer and when they are exogenously expressed they transform human cells. However no study to date has detected endogenous MDM2 protein isoforms. Studies with exogenous expression of splice variants have been carried out with *mdm2-A* and *mdm2-B*, but the *mdm2-C* isoform has remained virtually unexplored. We addressed the cellular influence of exogenously expressed MDM2-C, and asked if endogenous MDM2-C protein was present in human cancers. To detect endogenous MDM2-C protein, we created a human MDM2-C antibody to the splice junction epitope of exons four and ten (MDM2 C410) and validated the antibody with *in vitro* translated full length MDM2 compared to MDM2-C. Interestingly, we discovered that MDM2-C co-migrates with MDM2-FL at approximately 98 kDa. Using the validated C410 antibody, we detected high expression of endogenous MDM2-C in human cancer cell lines and human cancer tissues. In the estrogen receptor positive (ER+) *mdm2* G/G SNP309 breast cancer cell line, T47D, we observed an increase in endogenous MDM2-C protein with estrogen treatment. MDM2-C localized to the nucleus and the cytoplasm. We examined the biological activity of MDM2-C by exogenously expressing the protein and observed that MDM2-C did not efficiently target p53 for degradation or reduce p53 transcriptional activity. Exogenous expression of MDM2-C in *p53*-null human cancer cells increased colony formation, indicating p53-independent tumorigenic properties. Our data indicate a role for MDM2-C that does not require the inhibition of p53 for increasing cancer cell proliferation and survival.

## Introduction

The oncogenic activity of MDM2 is associated with over-expression of the MDM2 protein in human cancers [1–3]. While some MDM2 isoforms can form a direct physical association with p53 others do not [3,4]. MDM2 regulates the p53 protein through proteasomal-mediated degradation [5–10] and transcriptional repression [5,11–14]. This p53-mediated oncogenic activity of MDM2 is best referred to as MDM2 canonical functions. However, MDM2 is also known to possess p53-independent functions [15–19] that are best referred to as MDM2 non-canonical functions. 

The over-expression of MDM2 due to a single nucleotide polymorphism of thymine to guanine (T to G) at position 309 of the *MDM2* gene (*mdm2* SNP309) is associated with increased cancer incidence and aggressiveness [20–23]. This *MDM2* SNP309 nucleotide change increases the binding affinity for the constitutive transcription factor, Sp1 [21]. Cells homozygous for the G/G *mdm2* SNP309 have enhanced *mdm2* transcription and high MDM2 protein levels. MDM2 over-expression in cancers is often accompanied with the over-expression of alternatively spliced *mdm2* transcripts [3,24–28]. Over 40 alternatively and aberrantly spliced human *mdm2* transcripts have been reported, however not all are the bone fide result of alternative splicing events [29]. Not withstanding, the *mdm2* splice variants represent potential diversity that agrees with the findings of the Encyclopedia of DNA Elements (ENCODE) Project Consortium. ENCODE highlights previously unrecognized candidate regulatory elements, and encoded messages, in the human genome [30]. The diversity of *mdm2* spliced messages encoded from two independent promoters has the capacity to increase the human cancer proteome [31]. It is therefore not surprising that *mdm2* SNP309 cells demonstrate increased diversity in their alternatively spliced *mdm2* transcripts with substantial expression of the *mdm2-C* transcript [32]. 

Although over 40 *mdm2* alternatively spliced transcripts have been identified [29], only five, (*mdm2-A* through *mdm2-E*) have been shown to express protein *in vitro* [3]. The expression of these five *mdm2* transcripts causes NIH3T3 cells to form tumor-associated foci [3]. However, only two protein isoforms, MDM2-A and B, have been extensively studied for their biological functions. The exogenous expression of MDM2-A [33,34], or MDM2-B in mice [35], increases tumor formation in a *p53*-null, or p53-compromised background causing an altered tumor spectrum. However, the biological function of MDM2-C remains undetermined.

We asked if cancer cells expressing high levels of *mdm2-C* mRNA expressed endogenous MDM2-C protein. We hypothesized that high *mdm2-C* transcript levels encoded from the G allele *mdm2* SNP309 in human cancers would result in high levels of endogenous MDM2-C protein and would confer oncogenic functions. Cells with MDM2 over-expression via the G/G *mdm2* SNP309 have stable p53 protein, which is co-localized with p53 on the chromatin [14]. Thus, we hypothesized that MDM2 over-expression via the G/G SNP309 might produce an MDM2-C protein isoform that would not degrade p53. Therefore, we set out to determine the cellular function of exogenously expressed MDM2-C. We also asked if cancer cells expressing high levels of *mdm2-C* mRNA, also expressed endogenous MDM2-C protein. 

Endogenous expression of MDM2-C protein has never been detected due to the absence of antibodies that specifically detect the MDM2 isoforms made from the alternatively spliced mRNAs. The *mdm2-C* transcript does not contain exons 5 through 9, which encodes a part of the p53-binding domain. We created a specific antibody designed to detect the amino acids encoded by MDM2-C flanking exons 4 and 10, which we named C410. Using this MDM2 antibody we observed high basal levels of endogenous MDM2-C protein in various MDM2 over-expressing cancer cell lines and tissues. We also observed that, in the presence or absence of p53, exogenously expressed MDM2-C promotes increased colony formation. Taken together, our results indicate that endogenous MDM2-C is expressed in cancers and that MDM2-C functions independently of p53 to promote tumorigenesis. 

## Results

### MDM2 over-expressing cells have high levels of mdm2-C transcripts

Many human cancer cell lines over-express MDM2 protein and have been used for previous MDM2 studies [14,21,32,36,37]. We used these cell lines to examine the ratio of *mdm2-C* transcripts to full-length *mdm2* transcripts. The cell lines examined include two high MDM2 expressors: SJSA-1 cells with wild-type *p53* and over-expression of MDM2 due to *mdm2* gene amplification (and the *mdm2* SNP309 T/T alleles) and the MANCA cells with wild-type *p53* and over-expression of MDM2 from the *mdm2* SNP309 G/G alleles. They also include two low MDM2 expressors: the K562 cell line that is *p53*-null and has an *mdm2* SNP309 T/G alleles and the ML-1 cells with wild-type p53 and the *mdm2* SNP309 T/T alleles. 

**Figure 1:**
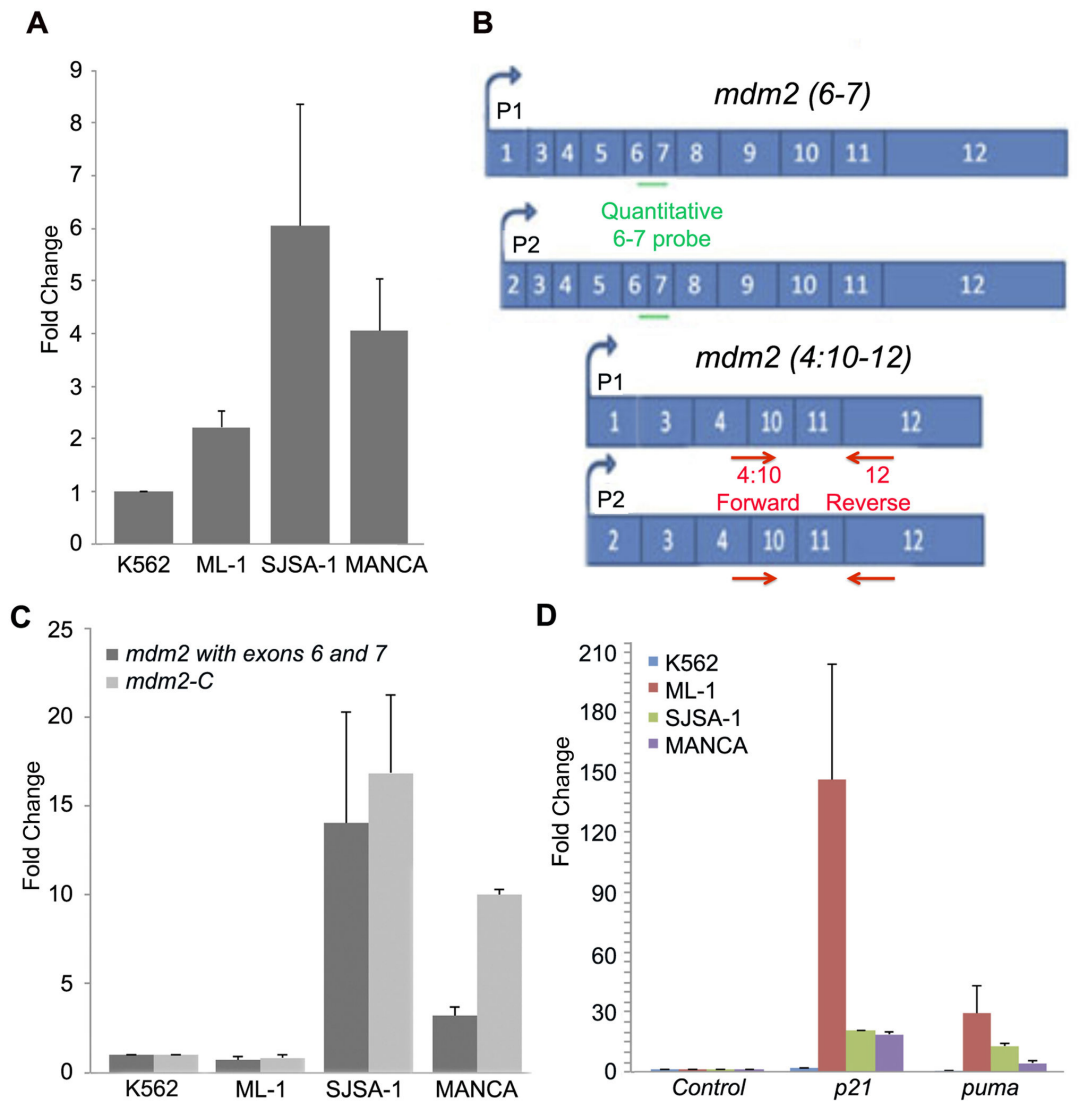
An *mdm2* splice variant transcript, *mdm2-C* is highly expressed in MDM2 over-expressing cells. **A**. Quantitation using Image J after northern blot analysis of RNA from untreated MANCA, SJSA-1, ML-1 and K562 cell samples. An *mdm2* DNA probe to exon 12 was PCR generated and radiolabelled with α ^32^P dCTP. Transcript levels were compared to K562 for basal expression and normalized for RNA levels to *gapdh*. An average of four independent experiments is shown. Error bars indicate standard error. **B**. Schematic of *mdm2* messages detected using a Taqman probe for exons 6 and 7 (6-7 probe) or forward primer for 4:10 and reverse primer for 12 (4:10-12 primer) for *mdm2-C*. **C**. qRT-PCR with 4:10 forward and exon 12 reverse vs. Taqman probe to exon 6 and 7 of *mdm2* were performed to detect *mdm2-C* and *mdm2* (with exons 6 and 7) transcripts. The *mdm2-C* transcripts were detected via Syber Green and *mdm2* (with exons 6 and 7) transcripts were detected via Taqman technology. Transcript levels were compared to K562 for basal expression and normalized for RNA levels to *gapdh*. An average of three independent experiments is shown. Error bars indicate standard error. **D**. qRT-PCR of RNA using Taqman technology from p53 target genes, *p21* and *puma* after DNA damage treatment with 8μM etoposide for 3 hours. Data from each cell line is presented as normalized to its own untreated control sample for fold activation and normalized for RNA levels to *gapdh*. An average of three independent experiments is shown. Error bars indicate standard error.

Transcription of *mdm2-C* was assessed by quantitative reverse transcription polymerase chain reaction (qRT-PCR) and northern blot analysis (see Figure 1 and Figure S1). Quantitation of total *mdm2* transcripts, by northern blot analysis, showed the highest level of *mdm2* transcripts in SJSA-1 cells, which contain 25 copies of the *mdm2* gene [37]. A high level of *mdm2* transcription was also observed in MANCA cells. To specifically quantify transcripts containing exons 6 and 7, we carried out qRT-PCR with a quantitative probe to the exon 6-7 junction (Figure 1B see probe in green and Figure 1C black bars). To specifically quantify *mdm2-C* transcripts, we used a forward PCR primer designed to recognize the exon 4, and exon 10, junction (called 4:10) and a reverse primer to exon 12 (Figure 1B, see primers in red, and Figure 1C grey bars). In MANCA cells, we detected low-level expression of the *mdm2* transcript containing exons 6 and 7, from here on referred to as a *mdm2* full-length transcript (Figure 1C). Importantly, MANCA cells had high-level expression of the *mdm2-C* transcript resulting in a lower ratio of full-length to *mdm2-C* transcript (Figure 1C, MANCA compare black to grey bar). This was in contrast to *mdm2* transcription in SJSA-1 cells, which produced similar levels of *mdm2-C* and *mdm2* full-length transcripts (Figure 1C, SJSA-1 compare black to grey bars). Low levels of *mdm2* transcripts were produced in K562 cells and therefore these cells were used as the normalizer cell line. ML-1 cells showed similar *mdm2* transcript levels to K562. This indicated that over-expression of MDM2 protein in SJSA-1 cells and MANCA cells correlated with high levels of the *mdm2-C* transcript. Moreover, MANCA cells had the highest ratio of *mdm2-C* to full-length transcript. This outcome may be due to the *mdm2* SNP309 G/G genotype that drives transcription from the inducible P2 promoter*.*

In order to see if the *mdm2* full-length to *mdm2-C* transcript ratio influenced the p53 response, we compared the activation of two p53 target genes, *p21* and *puma*. We treated K562, ML-1, SJSA-1 and MANCA cells with etoposide to initiate a DNA damage response, and monitored *p21* and *puma* activation by qRT-PCR (Figure 1D). As expected, the *p21* and *puma* genes were not activated in *p53*-null K562 cells. In the SJSA-1 and MANCA cells, which have wild-type p53 and high MDM2, the *p21* and *puma* genes demonstrated compromised gene activation compared to the activation detected in ML-1 cells, with MANCA cells demonstrating the least p53 activity (Figure 1D). 

*Mdm2* alternatively spliced products such as *mdm2-B* increase after DNA damage [38–40]. To determine whether the *mdm2-C* transcript was induced by p53, cells treated with etoposide were analyzed by qRT-PCR for *mdm2-C*. The ML-1 cells responded to etoposide treatment with a robust increase in *mdm2-C* transcript levels (Figure S2, red bar). Compromised p53-mediated activation of *mdm2-C* was observed in the SJSA-1 and MANCA cells similar to compromised activation observed for *puma* (Figure S2 and Figure 1D). These data show that p53 transcriptional activity was compromised in the MDM2 over-expressing cells and their high basal levels of *mdm2-C* (especially in MANCA cells) was p53-independent. 

## Validation of MDM2-C Specific Antibody

**Figure 2:**
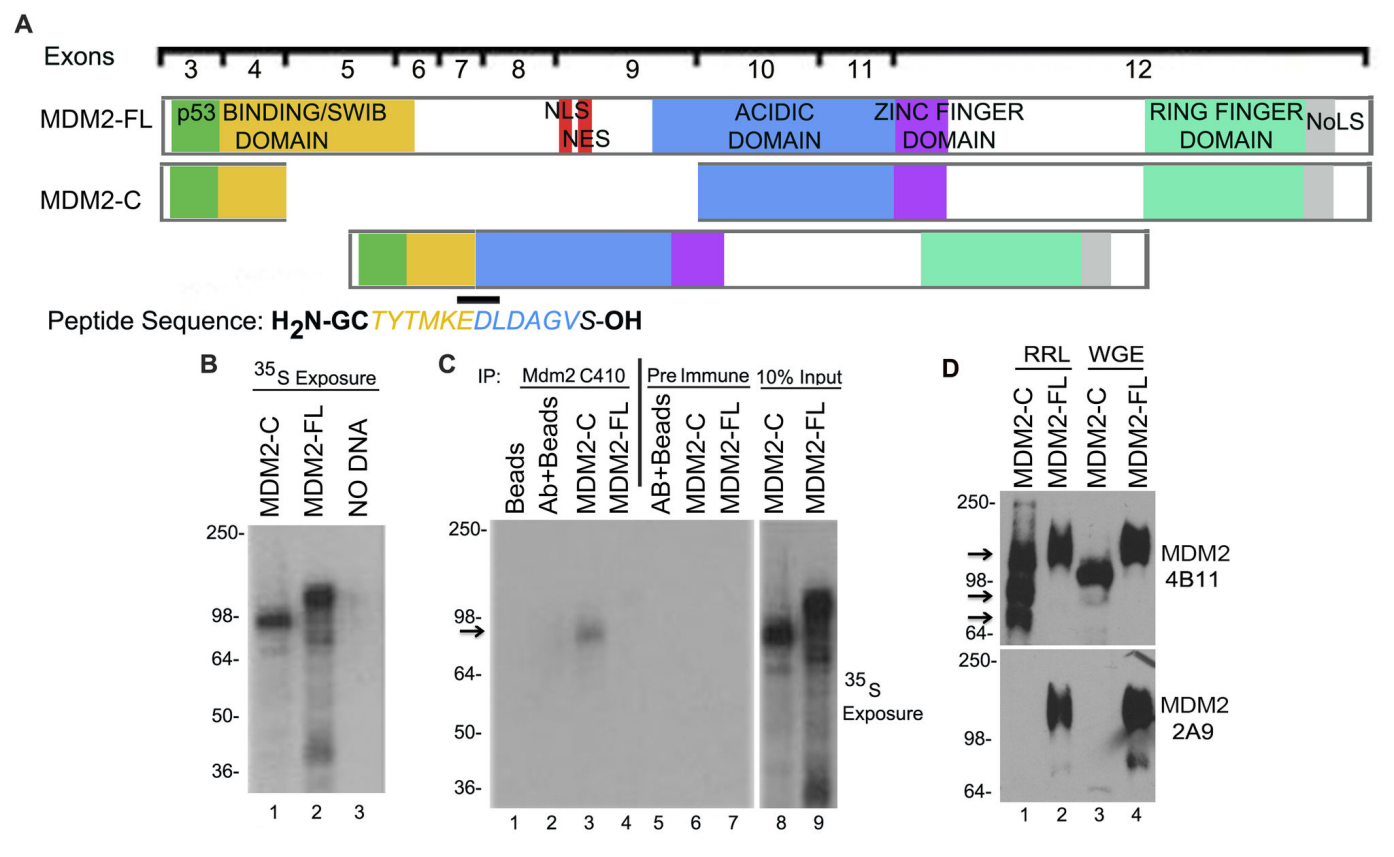
An MDM2-C specific antibody named C410, detects the MDM2-C protein. **A**. Schematic of full length MDM2 (MDM2-FL) and MDM2-C. The retained proteins’ biochemical functional domains are shown as color codes. The peptide sequence used as an immunogen containing the splice junction of human MDM2-C is shown. Glycine (G) and Cysteine (C) residues were added to the N-terminus of the peptide to facilitate conjugation to an immunoreactive protein, keyhole limpet hemocyanin (KLH). **B**. ^35^S methionine was used as a radioactivity source to label *in* *vitro* translated proteins. *In* *vitro* translations of pcDNA3-mdm2-FL and pcDNA3-P2mdm2-C using the TNT coupled wheat germ extract system. Resulting MDM2-FL and MDM2-C protein were electrophoresed on a10% SDS-PAGE in a 5:1:1 ratio. The gel was transferred to a nitrocellulose membrane and exposed to film for significant MDM2-C protein product detection. Wheat germ lysate without DNA was used as a negative control. **C**. Immunoprecipitation of ^35^S methionine radioactive-labeled *in* *vitro* translated MDM2-FL and MDM2-C proteins using MDM2 C410 and pre-immune polyclonal serum antibodies. Protein ratios as shown in **B** were used in the pull down assay. Samples were electrophoresed on a 10% SDS-PAGE gel transferred to a nitrocellulose membrane and exposed to film for protein detection. This is representative of three independent experiments. D. *In* *vitro* translated protein made in rabbit reticulocyte lysate (RRL lanes 1 and 2) were compared to protein translated in wheat germ extract (WGE lanes 3 and 4). These proteins were detected with either antibody 4B11 (top panel) or 2A9 (bottom panel). HRP-conjugated anti-mouse and anti-rabbit were used as secondary antibodies.

To determine if the high level of *mdm2-C* transcripts in MDM2 over-expressing cells was translated into protein, we generated an MDM2-C specific polyclonal antibody. We immunized rabbits with a peptide sequence containing the amino acid sequence of the human exon 4 to 10 splice junction (Figure 2A). The peptide was highly immunogenic and the antibody was validated for specificity using *in vitro* translated MDM2 proteins in wheat germ extract. Both MDM2-C, and MDM2-FL, can be translated *in vitro* into protein [3,41]. The addition of ^35^S methionine into the system enabled us to rapidly detect the specifically labeled MDM2 proteins using autoradiography (Figure 2B). The ^35^S methionine labeled MDM2-C and MDM2-FL proteins migrated on SDS-PAGE at sizes previously determined (described as higher than the predicted sizes of 36 kDa and 55 kDa, respectively) [41,42]. The slower mobility of MDM2 on SDS-PAGE has previously been attributed to the large acidic domain of the protein[42]. 

 To further validate the specificity of the MDM2-C antibody, we carried out an immunoprecipitation experiment of the wheat germ extract *in vitro* translated ^35^S radiolabeled MDM2-C and MDM2-FL protein products. The MDM2 C410 polyclonal antibody pulled down MDM2-C (Figure 2C, compare lanes 3 and 8) but not MDM2-FL (Figure 2C, compare lanes 4 and 9). Furthermore, MDM2-C and MDM2-FL *in vitro* translated proteins were immunoprecipitated using the MDM2 C410 polyclonal antibody and analyzed by western blot with MDM2 monoclonal antibody mix. Only the MDM2-C protein was detected (Figure S3, compare lanes 2 and 3).

**Figure 3:**
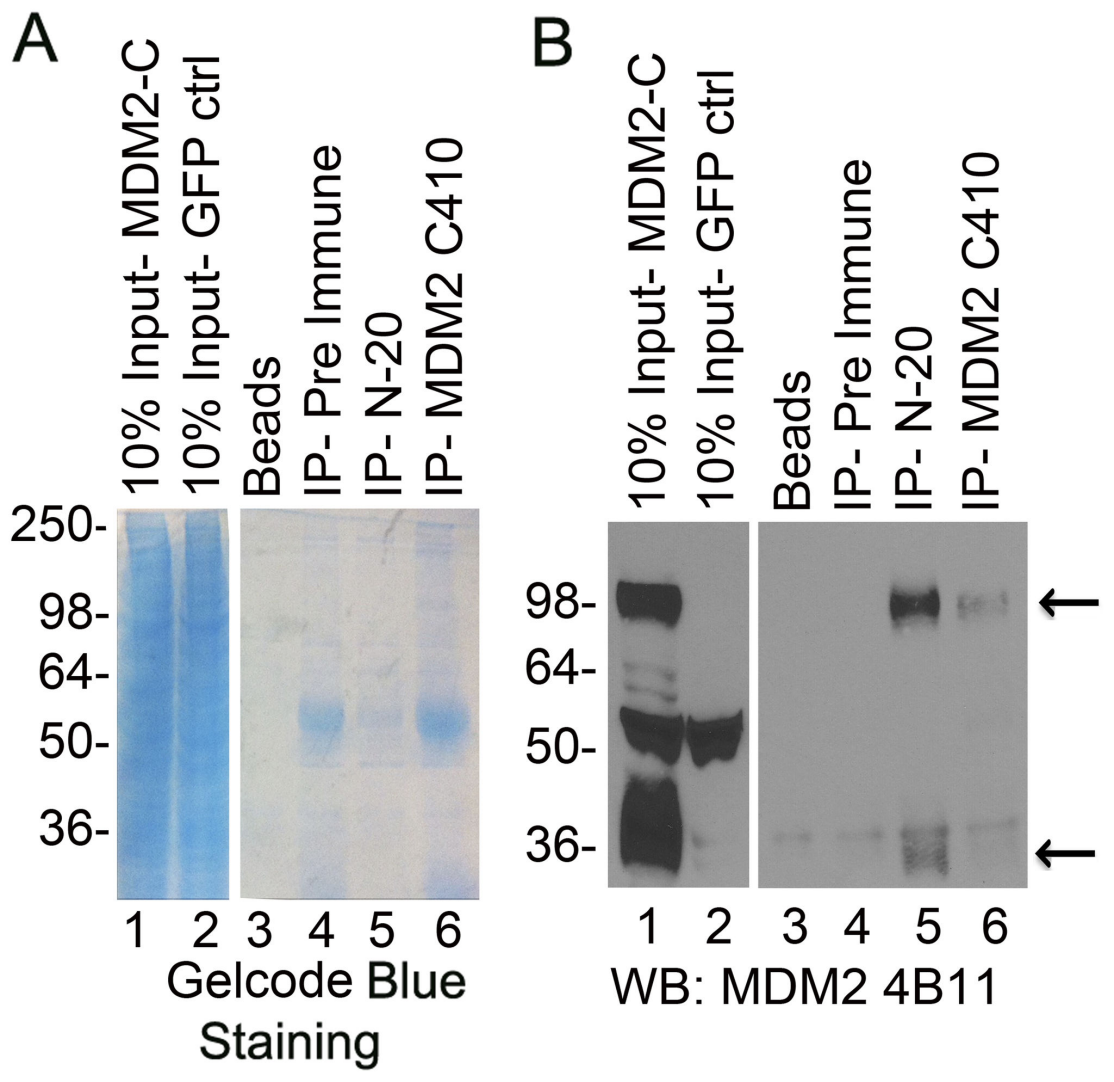
Human MDM2-C migrates at 98 kDa. Immunoprecipitation using MDM2 C410 and pre-immune polyclonal serum antibodies with HeLa *in* *vitro* translated MDM2-C extracts using pre immune sera, MDM2 C410 and N-20 polyclonal antibodies. Samples were electrophoresed onto a 10% SDS-PAGE gel in duplicate. **A**. One half was stained with coomassie blue for protein detection. **B**. Samples from half of gel were transferred to nitrocellulose membrane and MDM2 was detected with 4B11 monoclonal antibody. HRP-conjugated anti-mouse was used as secondary antibody. Arrows depict MDM2-C protein.

In order to more thoroughly address the differential migration of the MDM2-C and MDM2-FL, we compared the immuno-reactivity of *in vitro* translated proteins made in both rabbit reticulocyte lysate (RRL) and wheat germ extract (WGE) systems. When proteins are translated *in vitro*, they are post-translationally modified by the factors represented in the cell extract system. As predicted, the C-terminal monoclonal antibody 4B11 detected MDM2-C and MDM2-FL isoforms while the antibody 2A9, targeting the central region, detected only MDM2-FL (Figure 2D, compare top and bottom panels for lanes 1 and 3 versus lanes 2 and 4). Interestingly, the MDM2-C produced in the mammalian RRL system resulted in high expression of three differentially migrating species on SDS-PAGE electrophoresis with one form co-migrating with MDM2-FL at approximately 98 kDa (Figure 2D, compare lane 1 to lane 2). We further examined the potential post-translational modifications of MDM2-C in human cells (and the migration of MDM2-C at approximately 98 kDa) by utilizing a HeLa *in vitro* translation system from Pierce (Figure 3A and 3B). Importantly, in this system, we also detected a form of MDM2-C at approximately 98 kDa. Our data clearly indicate that some of the MDM2-C protein isoform produced in this human system co-migrates with MDM2-FL. Thus providing evidence that the detection of MDM2 by antibodies that can interact with the full-length and spliced variant isoforms will result in at least some co-migrating polypeptides on a western blot. Interestingly, using the HeLa system, we for the first time, detected some MDM2-C protein at the predicted molecular weight of 36 kDa (Figure 3B lane 1). 

### MDM2-C protein is expressed endogenously

**Figure 4:**
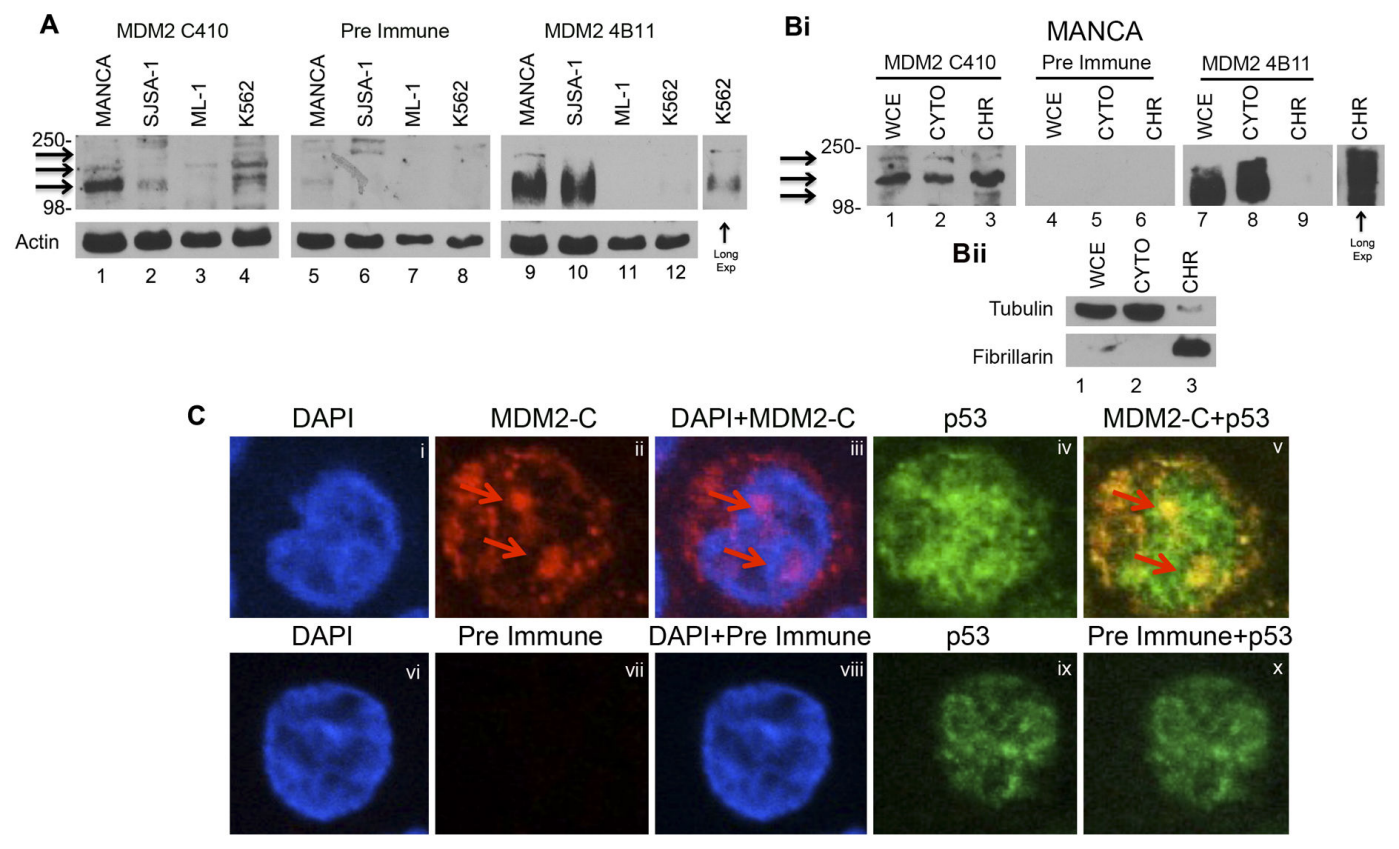
High expression of endogenous MDM2-C protein in G/G *mdm2* SNP309 MDM2 over-expressing cells. **A**. Western blot analysis of whole cell extracts from: MANCA, SJSA-1, ML-1 and K562 cells. MDM2-C protein levels were analyzed via MDM2 C410 polyclonal serum antibodies (C410, lanes 1-4) and total MDM2 was detected with the monoclonal 4B11 (lanes 9-12 and long exposure for lane 12). Actin was used as a loading control. Pre immune polyclonal serum was used as a negative control. HRP-conjugated anti-mouse and anti-rabbit were used as secondary antibodies. This is representative of three independent experiments. **Bi**. MANCA cells were lysed either as whole cell extracts (WCE) or into cellular compartments- cytosolic (CYTO) and Chromatin (CHR). Proteins were detected as in A. MDM2-C protein levels were analyzed via MDM2 C410 polyclonal serum antibodies (C410, lanes 1-3) and total MDM2 was detected with the monoclonal 4B11 (lanes 1-9 and long exposure for lane 9). **Bii**. Tubulin and Fibrillarin were used to show efficient cellular fractionation of extract. **C**. Spinning disk confocal microscopy of MANCA cells. Cells were fixed, permeabilized and incubated with p53, MDM2 C410 and pre-immune polyclonal serum antibodies. Slides were incubated with secondary Alexa-conjugated goat anti-rabbit and FITC-conjugated goat anti-mouse. DAPI was used to stain the cell nuclei. Pictures were taken at 60X magnification. Arrows indicate regions of Mdm2-C protein nuclear localization.

While alternatively spliced *mdm2* messages have been detected in cancers, there is no documentation on endogenously produced polypeptides from such messages. We compared the co-migrating nature of MDM2-C and MDM2-FL using the variable immunoreactivity of human cancer cell extracts probed with MDM2 C410 polyclonal antibody (for detection of MDM2-C) to the same extracts probed with the MDM2 pan-reactive mouse monoclonal 4B11 antibody (for the detection of MDM2-C and MDM2-FL). High levels of MDM2 expression were observed in MANCA and SJSA-1 cell extracts with 4B11 antibody (Figure 4A, lanes 9 and 10). MANCA cells produced the highest expression of MDM2-C when detected with C410 (Figure 4A, lane 1). The MDM2 C410 polyclonal antibody barely detected MDM2-C in ML-1 cells (Figure 4A, lane 3). However, it recognized MDM2-C protein in the cell extracts from K562 cells, which are G/T genotype cells, as well as in SJSA-1 cells that have a gene amplification (Figure 4A, lanes 2 and 4). The expression of MDM2-C in ML-1 cells and MANCA cells correlated with the basal transcription of *mdm2* full-length and *mdm2-C* (compare Figure 1C to Figure 4A). For reasons that remain unclear, a specific correlation between the *mdm2* transcripts and MDM2 protein isoforms was not apparent for SJSA-1 cells and K562 cells. This may have been due to the ratio of MDM2-FL protein to the spliced variant, and subsequent E3 ubiquitin ligase-mediated degradation of MDM2 proteins that reduced protein stability.

**Figure 5:**
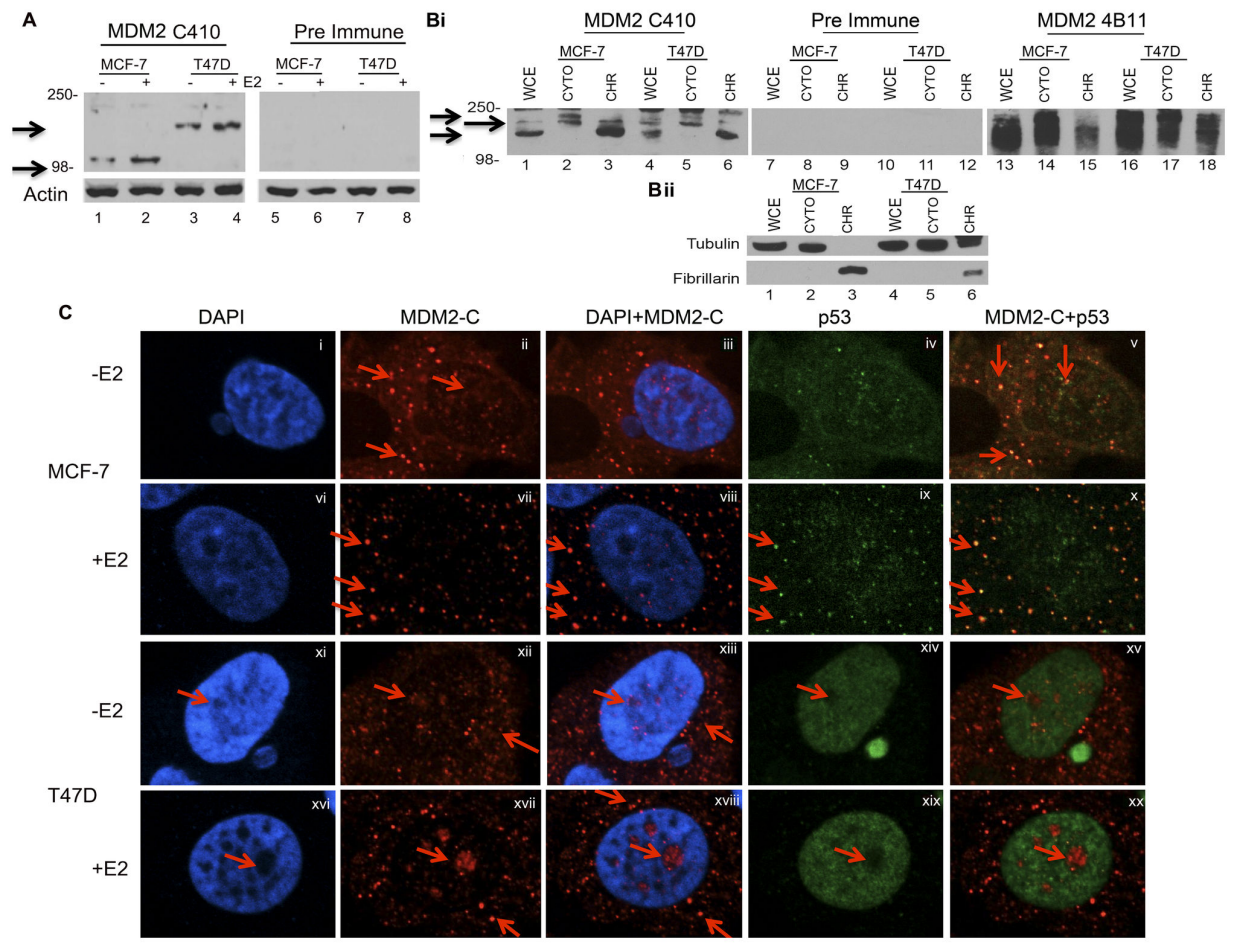
MDM2-C increases with estrogen treatment in ER+ Breast cancer cells and is located in the cytoplasm and nucleus. **A**. MCF-7 and T47D cells were grown and treated with 10nM estrogen (E2) for five days. Cells were lysed and proteins were analyzed via western blot. MDM2 C410 polyclonal serum anti-rabbit antibody was used for protein detection. Pre-immune polyclonal serum was used to detect background signal. HRP-conjugated anti-mouse and anti-rabbit were used as secondary antibodies. Actin was used as a loading control. This is representative of three independent experiments. **Bi**. MCF-7 and T47D cells were lysed for whole cell or chromatin fractionation extracts. 50μg of samples were resolved using 10% SDS-PAGE and MDM2 C410 polyclonal sera (lanes 1 - 6) and MDM2 monoclonal antibody, 4B11 (lanes 13 - 18) were used for protein detection. Pre immune was used to detect background signal (lanes 7 - 12). Secondary antibodies used were same as in A. **Bii**. Tubulin and Fibrillarin were used to determine fractionation purity. **C**. Spinning disk microscopy of MCF-7 and T47D cells. Cells were grown on coverslips and treated with 10nM E2 for five days. Cells were fixed and incubated with p53 antibody and Mdm2 C410 polyclonal antibody for protein detection. Pre-immune serum was used as a negative control for staining. Slides were incubated with secondary Alexa-conjugated goat anti-rabbit and FITC-conjugated goat anti-mouse. DAPI was used to stain the nuclei. Cells were visualized at 60X magnification. Arrows depict MDM2-C localization regions.

The high expression of MDM2-C in MANCA cells suggested that the protein would be clearly detectable in the cell compartments. We examined the localization of endogenous MDM2-C using cell fractionation methods (Figure 4B) and spinning disk confocal microscopy (Figure 4C). Using chromatin fractionation methods, we detected MDM2-C in the cytoplasm and on the chromatin (Fig. 4Bi, lanes 1-3) and the majority of the MDM2 isoforms detected with 4B11 were in the cytoplasm (Fig. 4Bi, lanes 7-9). Exogenously expressed MDM2-A and MDM2-B isoforms have previously been shown to exhibit nuclear localization [43] despite the fact that the regions encoding the MDM2 nuclear localization and export signals are spliced out. Similarly, MDM2-C, which also has these regions spliced out, localized to the cytoplasm and nucleus of these hematopoietic cells (compare DAPI and antibody stained area in Fig. 4Ci-iii probed with C410 and Fig 4Cvi-viii probed with pre-immune rabbit serum). MANCA cells express high levels of wild-type p53 protein [14], and we observed some co-localization of p53 and MDM2-C proteins in the cytoplasm and nucleus as indicated by yellow merge color (Fig. 4Cv). While the importance of co-localization is not immediately understood, we believe that it is an important observation. The red arrows point to strong antibody staining that represents a consistent observation of protein localized to discreet nuclear areas (Figs. 4Ciii and 4Cv). Since MANCA hematopoietic cells are small and do not have much cytoplasm, it was possible that what appeared cytoplasmic was on the periphery of the nucleus. Therefore, we examined MDM2-C localization in breast cancer epithelial cells and also observed nuclear and cytoplasmic MDM2-C localization (Figure 5C).

### MDM2-C increases with estrogen treatment and localizes to distinct punctate foci in the cytoplasm and nucleus of ER+ breast cancer cells

 MDM2 is elevated after estrogen treatment of estrogen receptor positive (ER+) breast cancer cells [36]. Furthermore, *mdm2* knockdown in estrogen treated breast cancer cells results in a decrease in proliferation, thus providing evidence for the importance of MDM2 in breast cancer cell growth. The over-expression of MDM2 is associated with increased *mdm2* spliced variant transcripts [3,24–28]. Therefore, we examined the expression of MDM2-C protein in two ER+ breast cancer cell lines, MCF-7 and T47D, possessing different genotypes for *mdm2* SNP30*9* (T/G versus G/G respectively) and *p53* (wild-type versus mutant respectively). We observed a slight increase in MDM2-C protein after estrogen treatment of MCF-7 and T47D cells (Figure 5A lanes 2 and 4). The reason for the difference in molecular weight of the MDM2-C isoforms might be due to differences in post-translational modifications in whole cell lysates. The pre immune polyclonal antibody serum was used as a background control for the MDM2 C410 antibody and resulted in practically no protein detection (Figure 5A lanes 5-8). In addition, in T47D cells, the *mdm2-C* message was increased two fold after estrogen treatment (data not shown). We fractionated the cells and examined cytoplasmic and chromatin fractions for MDM2-C protein (Fig. 5Bi for C410, rabbit pre immune and mouse monoclonal 4B11 reactivity and Fig. 5Bii for markers of fraction purity). The cytoplasmic fraction did not contain chromatin as evident by the absence of fibrillarin staining and each fraction showed some MDM2-C reactive protein (Fig. 5Bi lanes 2 and 5). Interestingly, the chromatin fraction from MCF-7 cells showed a strong MDM2-C reactive species and less 4B11 reactive protein (Fig. 5Bi, compare lanes 3 and 15).

 To determine if estrogen treatment influenced the localization of MDM2-C in MCF-7 and T47D cells, we carried out immunoflouresence. The MDM2-C in MCF-7 was detected in a pattern of diffuse and punctate cytoplasmic and nuclear localization before and after estrogen treatment (Figs. 5Cii and 5Cvii). Despite very little detectable p53 in the MCF-7 cells (Figs. 5Civ and 5Cix), the cytoplasmic p53 appeared to co-localize with the MDM2-C, as co-staining was detected as a yellow merge signal (Figs. 5Cv and 5Cx). 

The visualization of MDM2-C by immunoflouresence in the T47D cells, before and after estrogen treatment, displayed similar staining to MCF-7 cells (Fig. 5Cxii and 5Cxvii). However, T47D cells did not demonstrate co-localization of mutant p53 and MDM2-C (Fig. 5Cxv and 5Cxx). The reason for this difference in co-localization for MCF-7 cells and T47D cells might lie in the fact that these cell lines have wild-type versus mutant p53 proteins respectively. Many exogenously expressed MDM2 spliced isoforms do not interact with p53 [43,44] and are known to splice out the majority of the p53-binding domain [29]. We observed that T47D cells expressed predominantly nuclear mutant p53 (Fig. 5Cxiv and 5Cxix) while MCF-7 cells express low levels of cytoplasmic and nuclear wild type p53 [45]. 

### MDM2-C is highly expressed in liposarcoma and breast carcinoma tissues

**Figure 6:**
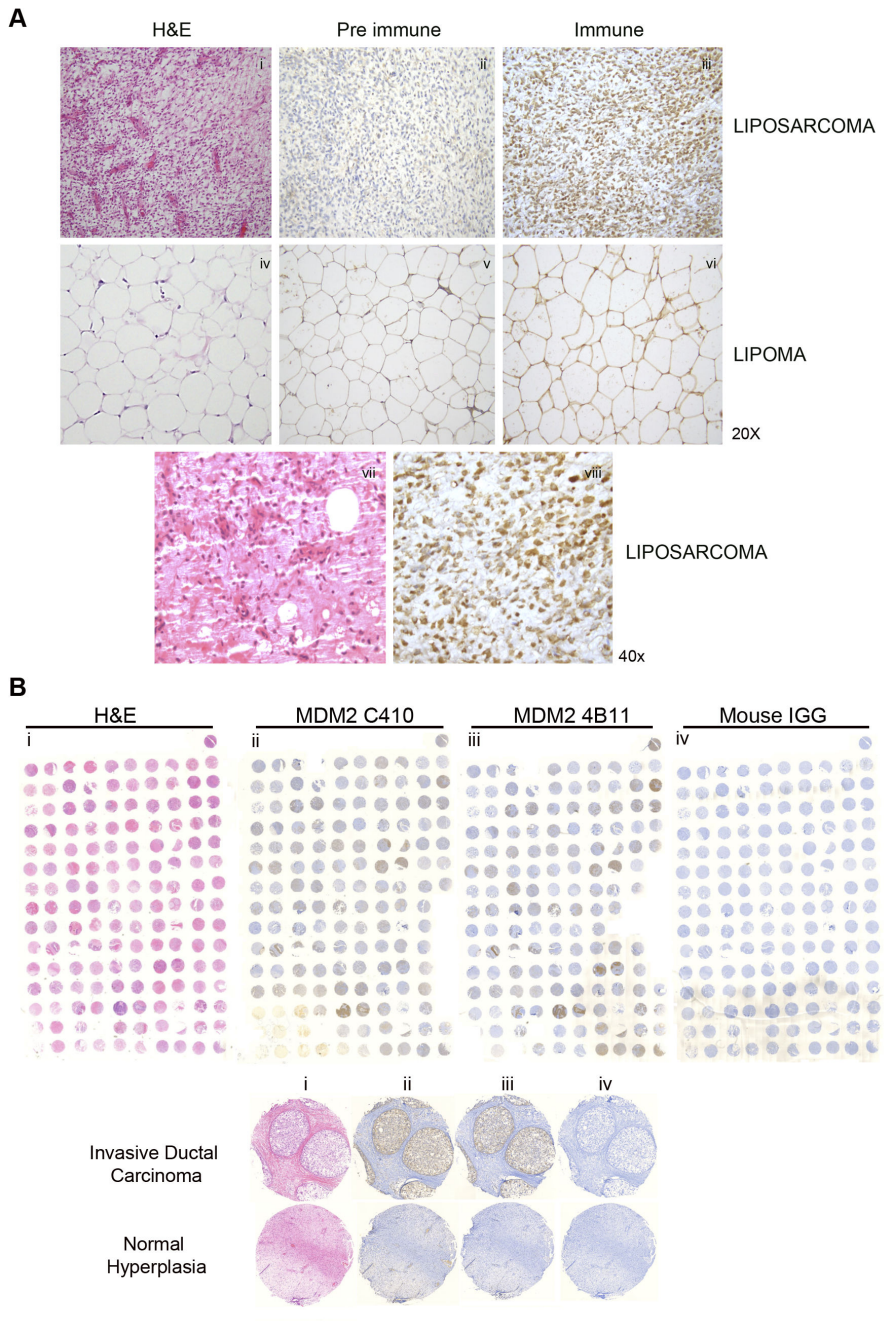
MDM2-C is highly expressed in liposarcoma and breast carcinoma tissues. **A**. Immunohistochemistry of lipoma and liposarcoma tissues using MDM2 C410 and pre-immune polyclonal serum antibodies. Biotinylated secondary antibody, ABC Reagent and DAB from Vector Labs were used. Pictures were obtained at 20X and 40x magnification. H&E refers to Hematoxylin and Eosin counterstaining. **B**. Breast tissue arrays (TMA-1008) were purchased from protein biotechnologies and automated histology was performed at the Molecular Cytology Core Facility at Memorial Sloan Kettering Cancer Center. Antibodies MDM2 C410 and MDM2 4B11 were used for staining. Mouse IGG was used for negative staining. H&E refers to Hematoxylin and Eosin counterstaining.

 High MDM2 expression is often observed in liposarcomas and is currently used as a cancer diagnostic biomarker [46–49]. We were interested in examining if the C410 antibody would be useful to examine MDM2-C as a liposarcoma biomarker in patient samples. We used immunohistochemistry (IHC) to test for MDM2-C expression in human liposarcoma tissue from human patient samples. For two out of three sample sets analyzed, positive staining for MDM2-C was observed (representative image shown in Figure 6A). The pre immune serum detected low background staining and when we examined lipoma tissues, only low positive MDM2-C staining was detected. We have begun similar studies using breast cancer tissue micro-arrays and have detected MDM2-C in invasive ductal carcinomas (Figure 6B). Using immunohistochemistry, we observed a similar staining pattern of positive tissues with MDM2 C410 and MDM2 4B11 antibody (Fig. 6Bii and 6Bii) while mouse IGG did not result in tissue staining (Fig. 6Biii).

### MDM-C binds to full length MDM2 and other proteins *in vivo*

Exogenously expressed MDM2-A and MDM2-B isoforms are known to interact with MDM2-FL [33,43]. To evaluate if endogenous MDM2-C in MANCA cells interacted with MDM2-FL and other cellular proteins, we performed protein pull-down assays with different MDM2 immunoreactive antibodies. We assessed the extent to which other proteins interacted with the endogenously expressed MDM2-C protein by culturing MANCA cells in media containing ^35^S methionine and examined the co-immunopreciptated radiolabeled proteins. Following MDM2-C protein pull down using the C410 antibody (as compared to the pre immune serum), we detected a number of proteins pulled down, with an MDM2 band slightly above 98 kDa detected in the western blot (Figure S4A). 

**Figure 7:**
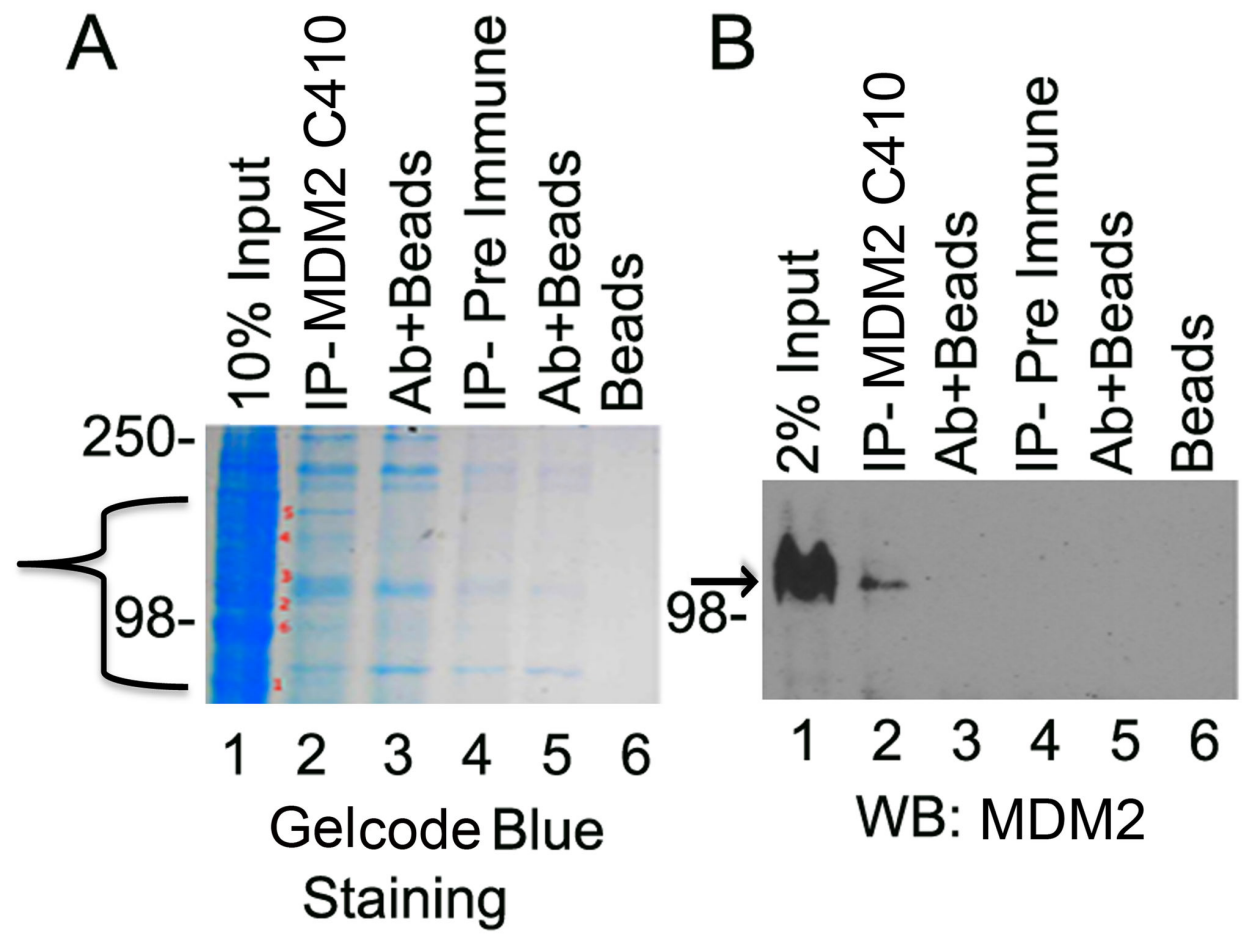
Human MDM2-C interacts with a variety of proteins *in* *vivo*. Immunoprecipitation from MANCA whole cell extracts using MDM2 C410 and pre-immune polyclonal serum antibodies antibodies. Samples were electrophoresed onto a 10% SDS-PAGE gel in duplicate. **A**. One half was stained with coomassie blue for protein detection and protein bands from A were excised for analysis via LC/MS/MS. **B**. Samples from half of gel A were transferred to nitrocellulose membrane and MDM2 protein was detected using the MDM2 monoclonal antibody mix (4B2, 2A9, 4B11). **A**. Bracket represents region where bands were cut out for LC/MS/MS analysis. **B**. Arrows depict MDM2-C protein.

The observation of many proteins co-immunoprecipitated with MDM2-C was also detected with non-radioactive proteins (Figures 7A and 7B). As described earlier, MDM2-C produced in the Pierce HeLa in vitro transcription translation kit migrated at approximately 98 kDa (Figures 3A and 3B, lanes 1-6). Importantly, the 4B11 antibody detected MDM2-C at 98 kDa and for the first time also at the predicted size of 36 kDa (Figure 3B, compare lanes 1 and 2). Moreover both 98 and 36 kDa forms were immunoprecipitated by the polyclonal C410 and N-20 antibodies (Figure 3B lanes 5 and 6). This strongly suggests that the C410 immuno-reactive MDM2-C isoform detected in MANCA cell extracts co-migrates with MDM2-FL at approximately 98 kDa. The SMP14 antibody epitope of recognition lies within the MDM2 nuclear localization and nuclear export signals absent in MDM2-C [29]. Interestingly, western blot detection using MDM2 C410 polyclonal serum antibody demonstrated that the SMP14 antibody co-immunoprecipitated MDM2-C isoforms that migrated at sizes above 98 kDa (Figure S4B lane 2- MDM2 C410 western blot). The MDM2-C form migrating above 98 kDa is proposed to be highly post-translationally modified forms of the MDM2-C protein. 

We made a preliminary identification of some of the radiolabelled proteins immunoprecipitated with MDM2 C410 antibody from MANCA cells, by performing liquid chromatography followed by tandem mass spectrometry (LC/MS/MS) for six predominant gel band regions observed after coomassie blue staining of the gel with non-radioactive samples in Figure 7A (and Table S1). LC/MS/MS identified a number of peptides, which corresponded to proteins with biochemical functions such as: RNA splicing, transcription, translation and metabolism. Based on “scaffold analysis” of LC/MS/MS with a stringency of at least five peptides identified with 95% probability, we identified 35 proteins (see Table S1 for protein sequence tags and accession gene symbols). Examples of such proteins included: Nucleolin, Heat Shock Cognate 71 Protein (HSP7C), Heat Shock protein HSP90β (HS90B), probable ATP-dependent RNA helicase (DDX5), and protein KIAA1967 (K1967). However, whether the proteins pulled down maintain direct or indirect interactions with MDM2-C, and the impact of MDM2-C binding on these proteins *in vivo* is not known. 

### MDM2-C does not function in the canonical proteasome MDM2 pathway

**Figure 8:**
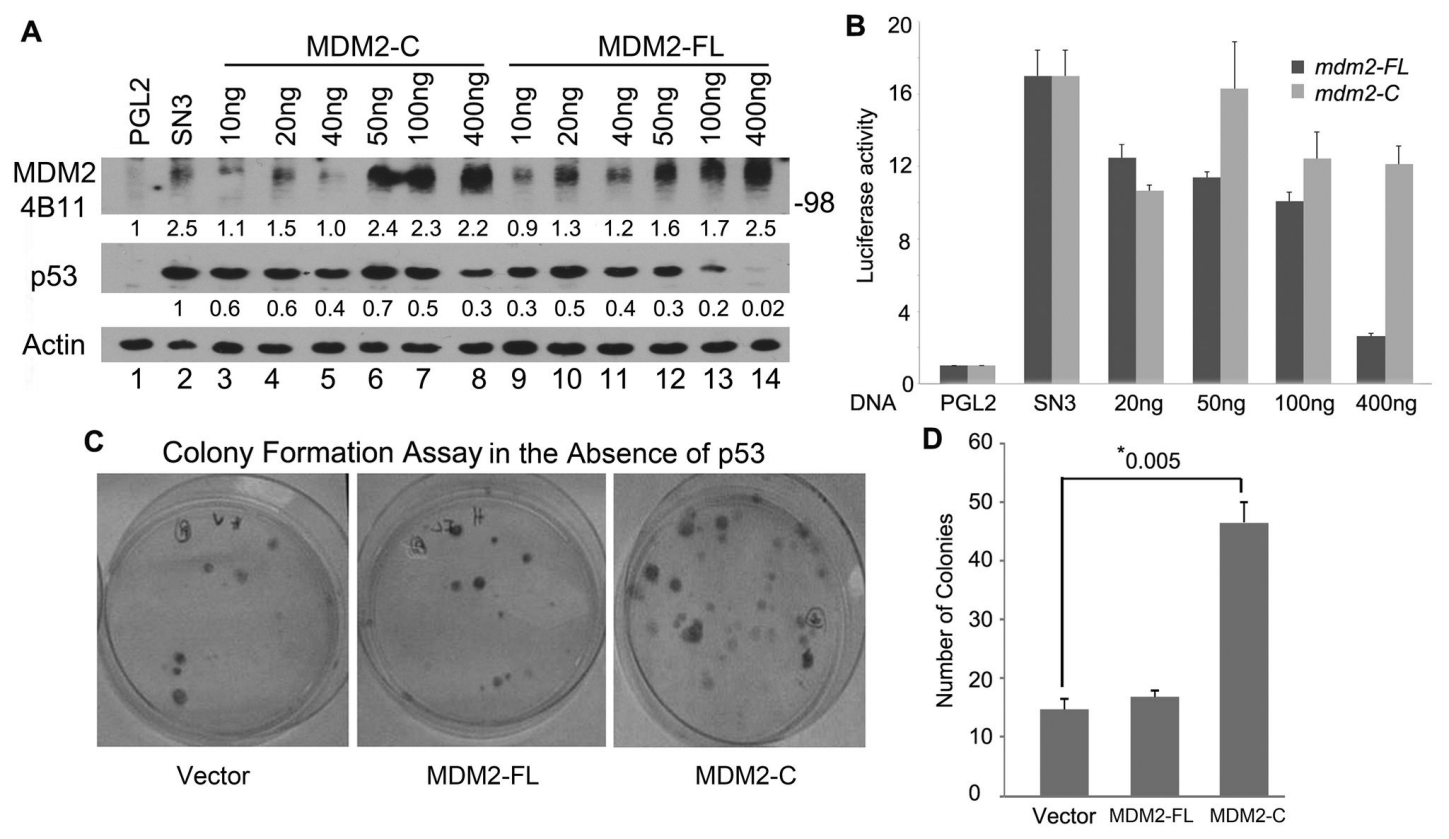
MDM2-C has p53-independent transformation activity. **A**. Analysis of exogenously expressed MDM2-FL or MDM2-C in H1299 cells with simultaneous expression of p53. Increasing amount of pcDNA3-*mdm2-FL* and pcDNA-P2mdm2*-C* plasmid DNAs were transiently transfected with a constant amount of SN3 plasmid (p53) into H1299 *p53-*null cells. PGL2 Basic plasmid was used as a DNA normalizer. Cells extracts were prepared 48 hours after transfection and analyzed for protein expression by western blot analysis using the Mdm2 monoclonal antibody mix (4B11) and p53 monoclonal antibody mix (240, 1801, 421). Actin was used as a loading control. HRP-conjugated anti-mouse and anti-rabbit were used as secondary antibodies. This is a representative of three independent experiments. **B**. 48 hours after transfection, cells were lysed and protein extracts were utilized in a luciferase assay reaction. Samples were compared to PGL2 Basic plasmid and normalized for amount of protein. An average of three independent experiments is shown. Error bars represent standard error. **C**. Colony formation assay in H1299. Cells were transiently transfected with the plasmid pBaBe-puro-*mdm2-FL* or pBaBe-puro-*mdm2-C* and after 24 hours, 2000 cells were plated into media (RPMI with 2ug/ml puromycin). Cells were allowed to grow for 3 weeks. Picture represents one experiment. Two experiments were carried out in duplicates. **D**. Colony counts after colony formation assay. An average of two experiments carried in duplicates is shown above. Error bars represent standard error. * Asterisks represent p-value compared to vector control.

Prior work in BJ cells (human fibroblasts) showed that exogenously expressed MDM2-A or MDM2-B isoforms decrease cell proliferation with no associated degradation of the p53 protein [28]. To determine the influence of MDM2-C on cellular proliferation, we exogenously expressed MDM2-C in *p53*-null H1299 cells in the presence and absence of exogenously expressed p53. An increase in co-migrating 98 kDa MDM2-C protein (Figure 8A, lanes 3-8) or MDM2-FL (Figure 8A, lanes 9-14) was detected with increasing amounts of *mdm2-FL* or *mdm2-C* expressing plasmid DNA. The image J values are shown in Figure 7A underneath the MDM2 4B11 and p53 probed blots. Importantly with MDM2-C expression, the p53 protein levels did not substantially decrease (Figure 8A, lanes 3-8, middle panel-p53 with image J values normalized to lane 2). This was in direct contrast to what was observed with the increased addition of *mdm2-FL* expressing plasmid, which resulted in substantial decreased p53 protein levels (Figure 8A, lanes 9-14, middle panel-p53 with image J values normalized to lane 2). This was seen most strikingly when the highest concentration of the *mdm2-FL* transfected plasmid was compared to *mdm2-C* (Figure 8A lane 8 vs. lane 14, middle panel – p53 levels). Image J quantification of the p53 signal showed approximately 10 fold less p53 protein in MDM2-FL expressing cells as compared to MDM2-C expressing cells. 

MDM2-A and MDM2-B isoforms are known to bind to MDM2-FL, sequester it to the cytoplasm and promote p53 activation [33,50]. We tested the effect of MDM2-C on p53 transcriptional activity using a luciferase reporter assay in cells co-transfected with p53 and either MDM2-C or MDM2-FL. Increasing MDM2-FL protein reduced the p53 transcriptional activity, while increasing MDM2-C protein did not (Figure 8B). These data indicate exogenous expression of MDM2-C did not function in the canonical p53 degradation pathway and did not significantly inhibit p53 transcriptional activity. 

The fact that MDM2-C did not function in a canonical MDM2 pathway caused us to assess the effect of MDM2-C on p53-independent colony formation as an indicator of tumorigenic properties. Colony formation assays were performed in human *p53*-null H1299 lung carcinoma cells transiently transfected with plasmids to express MDM2-C or MDM2-FL protein [7]. Cells were seeded at low cell number into drug selection medium and allowed to grow for a period of two to three weeks. After the prolonged incubation time, cells expressing MDM2-C formed more colonies as compared to cells expressing MDM2-FL or vector control (Figure 8C). Quantification of the data showed that H1299 cells expressing MDM2-C protein had the greatest number of colonies (Figure 8D). Validation of MDM2-C and MDM2-FL protein expression was observed by western blot analysis from the transfected H1299 cells after puromycin selection (data not shown). 

## Discussion

We report here that in human cancer cell lines that over-express MDM2, we detect high expression of the *mdm2-C* transcript and MDM2-C protein. We have identified an immunogenic region of human MDM2-C protein, which is a peptide from the exon 4 to exon 10 splice junction. This peptide was used for the development of a rabbit polyclonal MDM2-C specific antibody that was able to specifically detect exogenously and endogenously expressed MDM2-C protein in human cell lines and tissues. We found that MDM2-C protein co-migrates with MDM2-FL at an aberrantly high molecular weight. The cause for MDM2 isoforms migrating higher than their calculated molecular weight has remained a highly debated area. There have been some suggestions that this is due to post-translational modification of MDM2. We detected three different isoforms of MDM2-C produced in RRL (that were not detected with central region antibody) indicating a high likelihood of variable post-translational modifications of the MDM2-C protein. This is especially important because while it has been documented that MDM2 isoforms run above their predicted molecular weight, it has not been clear how much of the change is dependent on variable post-translational modifications. Our data indicate that MDM2-C is differently post-translationally modified in RRL and WGE systems (Figure 2D) as well as in the human HeLa systems from Pierce (Figure 3B); and that MDM2-C can migrate at approximately 98 kDa, which is the same mobility as MDM2-FL (Figure 2D). This has great implications for analysis of MDM2 western blots and suggests that in human systems, it is difficult to identify the variable MDM2 isoforms in SDS-PAGE. The development of monoclonal antibodies to human MDM2-C and other MDM2 splice isoforms will assist in the detection of oncogenic biomarkers for multiple types of cancers. 

The functions of MDM2-A and MDM2-B isoforms have been determined using exogenous protein expression. However, few studies have been carried out on MDM2-C. MANCA cells expressed a high ratio of *mdm2-C* to *mdm2-FL* transcript and high levels of MDM2-C protein (Figures 1C and 4A). We identified high levels of *mdm2-C* transcript in SJSA-1 osteosarcoma cells that have 25 copies of the *mdm2* gene [37] (Figure 1C), but detected very little MDM2-C protein (Figure 4A). The reason for this may lie in the almost one to one ratio of *mdm2-C* to *mdm2-FL* transcripts. It is possible that the higher levels of MDM2-FL protein result in low MDM2-C protein levels because MDM2-FL is acting as an E3 ubiquitin ligase for MDM2-C. This may account for the difference in MDM2-C protein levels observed in different cell types as suggested by the comparison of MANCA and SJSA-1 cells. 

Mice humanized as G/G for the *mdm2* SNP309 in two murine *mdm2* alleles produce a two and a half fold increase in *mdm2* transcript. This causes an increased cancer risk and changes the mutant p53 associated tumor spectrum including mammary adenocarcinoma [23]. We observed that SJSA-1 cells and MANCA cells (which are both resistant to cellular stress) [32] showed compromised induction of *p21* and *puma* transcription after DNA damage (Figure 1D). We also saw high levels of MDM2-C transcript and protein in the mutant p53 expressing T47D breast cancer cell line (Figure 5) [45]. This suggests that the levels of individual *mdm2* transcripts influence the wild-type p53 response as well as p53-independent cell survival pathways. 

MDM2 is over-expressed in over 30% of sarcomas, 80% of well-differentiated liposarcomas (WDLPS), and 60% of myxoid liposarcomas [51]. Using immunohistochemistry, we observed high expression of MDM2-C in liposarcoma and breast carcinoma patient tissues, and less expression of MDM2-C in lipoma and normal breast hyperplasia tissues (Figure 6). This suggests that MDM2-C may be used as a prognostic biomarker for cancers that have gained an MDM2 oncogenic pathway.

Exogenously expressed MDM2 isoform proteins localize to the nucleoplasm [43] and cytoplasm [28,40]. We found endogenous MDM2-C protein localized to the cytoplasm and nucleus of human cancer cells by immunofluorescent staining (Figures 4 and 5). We detected MDM2-C as punctate, suggesting that unlike the other MDM2 isoforms, MDM2-C might function in an alternate pathway related to its novel type of cellular distribution. This distribution pattern requires further study. Specific immunoprecipitation of MDM2-C from MANCA whole cell extract followed by Liquid Chromatography with tandem Mass Spectroscopy (LC/MS/MS) analysis allowed for a preliminary identification of co-associated proteins involved in splicing and translation (work in progress). However, MDM2 sequence tags were not identified most likely due to extensive post-translational modification of the protein in our samples. We hypothesize that MDM2-C is performing a function that is distinct from other MDM2 isoforms, which relates to its binding partners and cellular localization. It is possible that MDM2-C influences the splicing and translation of factors involved in growth promotion. The MDM2-C protein isoform maintains the RNA binding region of full length MDM2 [52–55]. Future experiments are needed to examine the RNA binding ability of MDM2-C. It is possible that we detected MDM2-C interacting with RNA binding proteins solely because such proteins were bound to an RNA transcript attached to MDM2-C. An alternative is that the *mdm2-C* transcript also has gene expression regulatory properties [56,57].

Increased MDM2 expression causes the degradation of wild-type p53 in a proteasome-dependent manner that requires three domains of MDM2 [7–10,58–62]. Splice variant isoforms of MDM2 do not degrade p53 although they retain the RING finger domain [29]. Simultaneous expression of MDM2-C with wild-type p53 in the *p53-*null H1299 cells demonstrated that wild-type p53 was not efficiently degraded by MDM2-C. The reason for this may lie in the fact that MDM2-C is lacking portions of the p53-binding domain critical in facilitating the MDM2-p53 interaction, which is important for p53 degradation [63]. Most MDM2 variant isoforms documented are unable to bind to p53 [43,44]. Therefore it is not surprising that MDM2-C did not substantially induce p53 degradation and appears to function through a p53-independent pathway.

MDM2 can promote cell proliferation independently of p53 and serve as a cancer biomarker [36,64]. Our data support the involvement of MDM2-C in p53-independent cell proliferation. The exogenous expression of MDM2-C in *p53*-null H1299 cells resulted in increased colony formation. In the ER+ breast cancer cell lines, we also observed an estrogen-dependent increase in MDM2-C protein. This suggests that MDM2-C could be a survival factor for ER+ cells following exposure to estrogen. Examining the levels of MDM2-C in cancer tissues could help identify cancers that are driven by p53-independent pathways. Our data implicate that MDM2-C has roles in p53-independent activation of cell proliferation. Two major cancer related proteins that functionally interact with MDM2 and are involved in cell proliferation and cell cycle control, are E2F and RB [65–67]. Whether MDM2-C protein functions in the E2F/RB pathway, other growth pathways, or participates in the regulation of RNA processing are questions that remain to be addressed. Our findings with MDM2-C open up a new avenue to explore in terms of cancer prevention and prognosis. 

## Materials and Methods

### Plasmids

Human MDM2 expression plasmid, pcDNA3.1-F-hdm2 was a generous gift from Dr. Carol Prives; PGL2-Basic; *p21*-Luciferase (*p21* p53 responsive elements) [68]; SN3 (p53) [69]; pBabe-puromycin (pBabe-puro) plasmid was a generous gift from Dr. Xuejun Jiang. For HeLa *in vitro* translation we used vector pT7CFE1-CGST-HA-His-GFP (Pierce/ Thermo Scientific). 

### Plasmid DNA Cloning

pcDNA3-F-hdm2 was used as a swap backbone to generate the clone containing exons-2, 3, 4, 10, 11 and 12 for pcDNA3.1-P2mdm2-C plasmid. Restriction enzymes- BamHI and EcoRI (New England Labs) were used for this swap to sub-clone exons 2-12 for *mdm2-C* into the pcDNA3-F plasmid retaining just the end of exon 12. DNA sequences for *mdm2-FL* and *mdm2-C* from the pcDNA3.1 vector were sub-cloned with enzymes- BamHI and EcoRI (New England Labs), into the pBabe-PURO vector. The *mdm2-C* coding region was inserted into the TOPO vector via TA cloning and sub-cloned from the TOPO-*mdm2-C* plasmid using restriction enzyme, NdeI into the IRES pT7 vector plasmid pT7CFE1-CGST-HA-His-GFP. 

### Cell culture

The tissue culture cells used in this study were: K562, ML-1, MANCA, SJSA-1, H1299, T47D and MCF-7. K562, SJSA-1, H1299, MCF-7 and T47D cells were purchased from American Type Culture Collection (ATCC); ML-1 cells were a generous gift from Michael Kastan [70]; MANCA cells were a generous gift from Andrew Koff [71]. Tissue culture cells were grown in RPMI 1640 media (Mediatech) or DMEM media (CellGro), containing 10% fetal bovine serum (FBS, Gemini) and 50U/ml penicillin and 50µg/ml streptomycin-pen-strep (Mediatech). Estrogen treatments were done using phenol red-free DMEM and 10% charcoal stripped FBS.

### Drug treatments

Drugs used in this study include: DMSO, etoposide, estrogen (17β Estradiol, E2): all drugs were purchased from Sigma. Cells were treated for 3 hours at the concentration of 8µM of Etoposide and three or five days at 10nM E2. 

### RNA isolation and quantitative RT-PCR

Cells were centrifuged at 1,100 rpm for 6 minutes at 4°C, washed twice with ice-cold phosphate buffered saline (PBS), and cells were frozen at -80°C overnight. The RNA was isolated using QIAshredder columns and the RNeasy Mini Kit (Qiagen) following manufacturer’s protocol and the resulting RNA was stored at -80°C. 5µg of RNA was used for cDNA synthesis using the High Capacity cDNA Archive Kit reagents and protocol (Applied Biosystems), where the RT master mix (containing RT buffer, dNTPs, random primers and MultiScribe reverse transcriptase) along with the RNA was incubated at room temperature for 10 minutes and then at 37°C for 2 hours. cDNA was stored at -20°C. Gene transcripts were amplified by quantitative PCR (qRT-PCR) with primer probes for *puma* (Hs00248075_m1), *mdm2* (Hs00242813_m1), *p21* (Hs00355782_m1) and *gapdh* (4333764) from Applied Biosystems Assays on Demand primers. In addition, primers to *mdm2-C* (exon 4:10 Forward: GAAAGAGGATCTTGATGCTGGTGTA and exon 12 Reverse: GGGGGATTCATTTCATTGCATG- both in the 5’ to 3’ direction) were used in a Syber Green qRT-PCR reaction to quantify *mdm2-C* transcripts. 150ng of cDNA was combined with Taqman Universal Master Mix and qRT-PCR was carried out following the program: one cycle, 2 minutes, 50°C; one cycle, 10 minutes, 94°C; and 40 cycles, 15 seconds, 94°C and 1 minute 60°C in a 7500 Sequence Detection System (Applied Biosystems). 

### Preparation of Radioactive probe

Plasmid DNA was combined with Taqman Universal Master Mix and primers flanking regions of *mdm2* exons 12 (forward: TTCGTGAGAATTTGGCTTCCT, reverse: GGCAGGGCTTATTCCTTTTC- both in the 5’ to 3’ direction) for PCR in a GeneAmp 5400 (Perkin Elmer, Waltham, Mass). The amplification steps were as follows: one cycle, 5 minutes 50°C, 10 minutes, 94°C; 35 cycles, 30 seconds, 94°C, 30 seconds, 58°C, and 2 minutes 72°C; and 10 minutes, 72°C. The resulting PCR product was purified using the QiaQuick PCR Purification Kit (QIAGEN). 25ng of PCR template was used for Random labeling with the High Prime Random Labeling Kit (Roche) and Easy Tag α-^32^P dCTP (Perkin Elmer) was used as the radioactivity source. The resulting probe was cleaned using Sephadex^TM^ G-50 DNA Grade Nick Columns (GE Healthcare) and the count per minute (CPM) was measured using a Scintillation counter (Perkin Elmer)**.**

### Northern blot analysis

mRNA samples were purified from total RNA using the Oligotex mRNA Mini Kit (QIAGEN). Total RNA samples were separated using a 1.0% denaturing formaldehyde agarose gel followed by a 10X SSC capillary action transfer onto a BrightStar-Plus positively charged Nylon membrane (Ambion) no more than 6 hours. RNA was cross-linked to the membrane via Ultra Violet exposure at the optimal setting for 1 minute. The membrane was then pre-hybridized in formamide pre-hybridization/hybridization solution for 3 hours at 42°C after which the probe (heated at 95°C for 10 minutes and placed on ice to cool) was added to the membrane for hybridization over night. The membrane was then washed at different stringencies (2X SSC/ 0.1%; 0.2% SSC/ 0.1% SDS and 2X SSC). Autoradiography was achieved by exposing the membrane to Kodak BioMax XAR Films (KODAK) at room temperature and at - 80°C with screen.

### *In vitro* transcription/ translation

100-1000ng of linearized pcDNA3-*mdm2-FL* or pcDNA3-*mdm2-C* plasmid DNA was mixed with components of the wheat germ extract kit or rabbit reticulocyte extract kit (Promega) and allowed to sit at 30°C for 90 minutes. Radiolabelled ^35^S Methionine (Promega) was used as a radiolabel source. The resulting protein was resolved on a 10% polyacrylamide gel. HeLa Cell *In vitro* translation in 1-Step Heavy Protein IVT system (Piece/Thermo Scientific) -1-2μg of pT7CFE1-*mdm2-C* (or pT7CFE1-CGST-HA-His-GFP) was mixed with the components of the HeLa cells lysate *in vitro* translation system and placed at 30°C for 8-24 hours. The samples were centrifuged at 14,000 x g for 5 minutes and the supernatant was frozen at -80°C.

### Radioactive labeling of endogenous proteins

MANCA cells were grown in 10% FBS/RPMI (Mediatech) minus Methionine supplemented with > 1000mCi of ^35^S Methionine. Cells were collected and lysed after 24 hours. 

### Cellular protein extracts- Whole cell extracts

Cells were pelleted at 1,100 rpm for 6 minutes at 4°C, washed twice with ice-cold PBS and re-suspended in 1.5-2X packed cell volumes of 1X lysis buffer (0.1% TritonX-100, 50mM Tris.Cl PH-7.5, 15mM EGTA, 100mM NaCl, 1 mM PMSF, 8.5 μg/ml Aprotinin and 2 μg/ml Leupeptin). The cell suspension was vortexed at intervals for 30 minutes on ice to lyse the cells and centrifuged at 13,000 rpm for 20 minutes at 4°C. The supernatant was stored at -80°C. 

### Chromatin Fractionations

Cells were pelleted at 1100 rpm for 7 minutes at 4°C and washed three times with ice- cold phosphate buffered saline (PBS). Cells were suspended in Buffer A (10 mM HEPES pH 7.9, 10 mM KCl, 1.5 mM MgCl2, 0.34M Sucrose, 10% glycerol, 1mM DTT, 0.5 mM PMSF, 2µg/ml Leupeptin, 8.5µg/ml Aprotinin) + 0.1% Triton X-100. Incubated on ice 5 minutes. Spun down cells 3600 rpm for 5 minutes at 4°C. Spun down supernatant for an additional 5minutes at 13,000 rpm at 4°C to clarify (S1 Fraction). Washed pellet 2 times with Buffer A spinning down at 3600 rpm for 5min at 4°C. Resuspended nuclei pellet in Buffer B (3 mM EDTA, 0.2 mM EGTA, 0.5 mM PMSF, 2µg/ml Leupeptin, 8.5µg/ml Aprotinin). Incubated on ice 30 minutes with vigorous vortexing every 5 minutes. Spun down 4000 rpm for 5 minutes at 4°C. The supernatant is nuclear soluble proteins (S2 Fraction) and the pellet is enriched in chromatin. Washed pellet 2 times with Buffer B. Resuspended pellet (P3 Fraction) in RIPA buffer and sonicated 3 times 30 seconds/30 seconds rest on ice. Froze samples at -80°C. 

### Western blot analysis

Protein samples were prepared in 4X protein sample buffer (4X NuPAGE Lithium Dodecyl Sulphate buffer, 20mM DTT). Samples were heated at 70°C for 10 minutes after which 100mM Iodoacetamide (Sigma-Aldrich) was added to prevent re-ligation of disulfide bridges between polypeptides. All samples were separated via 10% SDS-PAGE followed by an electro-transfer to nitrocellulose membrane. The membrane was blocked in 5% non-fat milk solution of 1XPBS/ 0.1% Tween 20 and was probed overnight at 4°C. Washes were done with 1XPBS/0.1% Tween 20 solution. Secondary anti-mouse or anti-rabbit antibody (Sigma) was applied to the membrane for 1hr at room temperature and the membrane was washed three times. Protein signal was visualized by chemiluminescence using the Super signal kit (Pierce) and detected after exposure for autoradiography to Hyblot CL films (Denville Scientific).

### Antibodies

p53- mouse anti-human monoclonal supernatant antibodies mix- 1801, 421 and 240 (N-terminus, Central and C-terminus regions respectively) [72], DO-1- mouse anti-human monoclonal antibody (Santa Cruz); Mdm2- mouse anti-human monoclonal supernatant antibodies mix- 4B2, 2A9 and 4B11 (N-terminus, Central and C-terminus regions respectively) [42], SMP14- mouse anti-human monoclonal antibody (Sigma); N20- mouse anti-human polyclonal antibody (Santa Cruz); MDM2-C- rabbit anti-human polyclonal pre-immune serum and MDM2 C410 immune polyclonal serum antibody (Biosynthesis); Actin- rabbit anti-human polyclonal antibody (Sigma); fibrillarin (Abcam), tubulin (Sigma)**.**

### Immunoprecipitation

*In vitro* translated protein products or whole cell extracts of MANCA were incubated with 1-2μg (SMP14 or N20) or 5-10μl (serum) primary antibody (MDM2 C410 serum, pre-immune serum) in 1X lysis buffer (0.1% Triton-X w/v, 50mM Tris.CL (P.H 7.5), 15mM Ethylene Glycol Tetraacetic Acid-EGTA, 100mM Sodium Chloride-NaCL, 1mM Phenylmethanesulfonyl Fluoride-PMSF, 2μg/ml Leupeptin and 10.3μg/ml Aprotonin). The mixture was placed at 4°C over night. The next day, protein-A sepharose beads were added for 3 hours. The protein-Antibody-beads mixture was spun down at 3000 rpm for 2 minutes and washed 3X with 1X PBS/0.1% TWEEN 20. The complex was re-suspended in 30μl 1X Protein sample buffer with 20mM DTT. Samples were heated at 70°C for 10 minutes and 1mM Iodoacetamide (Sigma) was added and resolved on a 10% acrylamide gel. 

### Immunofluorescence

Cells were seeded onto glass cover slips at 10% confluency and after indicated treatments, washed once with 1X PBS and fixed with 4% paraformaldehyde in 1XPBS for 15 minutes at room temperature. MANCA cells were spun down onto poly-lysine covered cover slips and treated Slides were washed three times with 1X PBS, permeabilized with 0.5% Triton-X-100 in 1X PBS/1% FBS for 10 minutes at room temperature and washed three times with 1X PBS/1% FBS. The cells were incubated for 1 hour at room temperature with 1:200 dilution of rabbit anti-human MDM2 C410 serum or rabbit anti-human pre-immune serum, p53 mouse anti-human monoclonal DO-1. The slides were washed three times with 1XPBS/1% FBS, incubated with 1:200 dilution of Alexa-conjugated goat anti-rabbit (Invitrogen) and FITC-conjugated goat anti-mouse (Jackson ImmunoResearch) secondary antibodies, and washed again three times with 1XPBS/1% FBS. Coverslips were then mounted onto slides using Vectashield mounting medium containing 4’, 6-diamidino-2-phenylindole (DAPI) to visualize the nuclei. Images were collected with a Nikon fluorescent microscope at 60X magnification.

### Spinning disk confocal microscopy

Cells were visualized using spinning disk confocal microscope. All confocal images were captured with the 60X objective. Images were visualized and captured under different channels: rhodamine (red), FITC, and ultra violet light (Blue for DAPI). 

### Immunohistochemistry

Paraffin embedded slides were de-paraffinized with Xylene (2x 3 minutes) and Xylene:100% Ethanol (3 minutes). The slides were then hydrated in 100% Ethanol (2x 3 minutes, 95% Ethanol (3 minutes), 70% Ethanol (3 minutes) and 50% Ethanol (3 minutes). Slides were rinsed in water and placed in the antigen retrieval buffer (10mM Tris base, 1mM EDTA and 0.05% Tween 20 PH 9.0) and boiled for 20 minutes after the solution had come to boiling. The slides were rinsed in cold water and washed 3x 5 minutes in 1X wash buffer (1XPBS/0.025% Triton X-100). Slides were blocked in blocker solution from the Vectastain ABC kit (Vector Labs) for 1 hour at room temperature. The slides were rinsed in 1X wash buffer 3x 5 minutes. Primary antibody – C410 serum and pre-immune polyclonal serum were diluted to 1:100 in 1X PBS/1%BSA and incubated over night at 4°C. The next day, slides were rinsed 3x 5 minutes in 1X wash buffer. The secondary antibody mixture (Vector Labs) was diluted in blocking solution and incubated fro 45 minutes at room temperature. The slides were washed 3x for 5 minutes in the wash buffer. The slides were developed for approximately one minute per slide using the Peroxidase Substrate kit DAB (Vector Labs). The slides were rinsed with water immediately when staining began to develop. Slides were counterstained with Hams modified Hematoxylin and Eosin [73] and washed in water 2x 5 minutes. The slides were then dehydrated as follows: 2x 95% Ethanol, 2x 100% Ethanol and 2x 100% Xylene. The slides were then mounted with Permount solution [73] and visualized under the Nikon bright upright microscope and pictures are taken at 20X magnification. Breast tissue arrays were purchased from “Protein Biotechnologies Ramona, CA”. The “Molecular Cytology Core Facility” at Memorial Sloan Kettering Cancer Center performed histology.

### Transfection

Approximately 2-6x10^5^ cells were plated per well in 6-well plates or at 50% confluence into 10cm plates per transfection. Cells were treated with Lipofectamine 2000 (Invitrogen) as per manufacturers’ instruction. 10ng of SN3 plasmid and 10-1000ng *pcDNA3.1-Fhdm2* or 10-1000ng *pcDNA3.1-P2mdm2-C*, were co-transfected with 10ng of SN3 [69] plasmid and *p21-luciferase* plasmid [68]. *PGL2* plasmid was used as a DNA control.

### Luciferase assay

Cells were co-transfected with SN3, p21-luciferase reporter plasmid and pcDNA3.1-F*hdm2* or pcDNA3.1-P2*mdm2-C*. 48 hours after transfection, cells were lysed as stated above and assayed using the luciferase reporter assay (Promega) and measured via Luminoskan. Samples were normalized for the amount of protein.

### Trypan Blue Staining

Cells are trypsinized and spun down at 1100rpm for 5 minutes. The cell pellet was re-suspended in serum-free DMEM and 0.4% Trypan blue dye (Sigma) was added to the cells at a 1:6 dilution ratio. The dye and cells mixture was allowed to sit at room temperature for 5 minutes and the total numbers of cells as well as the number of blue cells were counted. 

### Colony formation assay

H1299 cells were co-transfected with or without 10ng of SN3 plasmid and 400ng of pBaBe-*mdm2-FL* or pBaBe-puro-*mdm2-C* plasmids. Five hours after transfection, the media was changed to complete RPMI/10%FBS or DMEM/10% FBS. 24 hours later cells were diluted and 2000 cells were plated into media with 2µg/ml puromycin on 10cm plates and allowed to grow for 2-3 weeks. Colonies were stained with methylene blue (Sigma-Aldrich) and counted. 

### Coomassie staining

Acrylamide gels were rinsed 3X with Mill-Q water for 15 minutes each and stained for an hour using Gel Code Blue (Thermo Scientific) as per manufacturer’s suggestions. 

#### Liquid Chromatography tandem Mass Spectrometry (LC-MS/MS)

After MDM2 C410 immunoprecipitation and coomassie staining of co-immunoprecipitated proteins on the SDS-PAGE, protein bands were excised and trypsin was utilized for sample digestion. All samples were subjected to LC-MS/MS and the resulting data was uploaded onto a proteome software program, scaffold 3. Scaffold 3 analyzes the MS/MS data and collates the information in a table format giving the identified peptides, their corresponding proteins, biological functions and classification based on biological function. The program identifies a protein based on the peptides observed and their frequency. The data obtained was put in a table format according to the frequency of peptides observed and the protein sequence tags detected. The analysis was performed by the Mass Spectrometry facility of the Proteomics and Microchemistry Core Facility at Memorial Sloan Kettering Cancer Center (MSKCC). 

## Supporting Information

Figure S1 **MDM2 over-expressing cells have high mdm2 transcript levels.** Northern blot quantitation of total RNA from untreated K562, ML-1, SJSA-1 and MANCA, cells. RNA samples were electrophoresed onto a 1% denaturing formaldehyde agarose gel and transferred to a nylon membrane. The northern blot was probed with an exon 12-specific probe for mdm2 and exposed to film for transcript detection. α ^32^P dCTP was used as a radioactivity source for the probe labeling. GAPDH was used as a normalizer for RNA levels. Relative mdm2 message for lanes 1 - 4 was an average of 1, 2.2, 6 and 4 respectively shown in Figure 1.
(TIF)

Figure S2 **Etoposide activated transcription of *mdm2* is compromised in MDM2 over-expressing cells.** qRT-PCR of RNA from *mdm2-C* genes using syber green technology after induction of p53 via 8μM etoposide for 3 hours. Each cell line was normalized to its own control sample for fold activation and *gapdh* for RNA levels. An average of three independent experiments is shown. Error bars indicate standard error.
(TIF)

Figure S3 **MDM2 C410 antibody is specific to MDM2-C protein.** Immunoprecipitation of *in* *vitro* translated MDM2-FL and MDM2-C proteins using MDM2 C410 and pre-immune polyclonal serum antibodies. MDM2 monoclonal antibody mix (4B2, 2A9, 4B11) was utilized for protein identification. Wheat germ lysate without plasmid DNA was used as a negative control. HRP-conjugated anti-mouse and anti-rabbit were used as secondary antibodies. This is a representative of three independent experiments. Arrow represents MDM2-C band.
(TIF)

Figure S4 **MDM2-C interacts with MDM2-FL and other cellular proteins *invivo*.** **A**. Immunoprecipitation of ^35^S Methionine radioactive labeled MANCA whole cell extracts using Mdm2 C410 and pre-immune antibodies. Samples were electrophoresed on a 10% SDS-PAGE gel, transferred to nitrocellulose membrane and exposed to film for protein detection. Arrows represent MDM2 protein bands.
**B**. Immunoprecipitation from MANCA whole cell extract using MDM2 antibody, SMP14. Samples were electrophoresed on a 10% SDS-PAGE gel, transferred to a nitrocellulose membrane and probed with MDM2 C410 polyclonal serum antibody. Immunoprecipitation was also performed with the MDM2 C410 and pre immune antibodies. Samples were electrophoresed on a 10% SDS-PAGE gel, transferred to a nitrocellulose membrane and probed with MDM2 4B11 monoclonal serum antibody. The membrane was sequentially re-probed with MDM2 2A9 monoclonal antibody. HRP-conjugated anti-mouse and anti-rabbit were used as secondary antibodies. Arrows represent MDM2-C interacting protein bands.
(TIF)

Table S1 **List of proteins identified by LC/MS/MS and their protein sequence tags.** After LC/MS/MS was carried out by the Memorial Sloan-Kettering Cancer Center Proteomics and Microchemistry Core Facility we used “scaffold analysis” of the identified peptides. We employed parameters with a stringency of at least five peptides with 95% probability, which identified 35 proteins.
(XLS)
