# Supplementary figures and images for: Endogenous Human MDM2-C Is Highly Expressed in Human Cancers and Functions as a p53-Independent Growth Activator
Source: PLoS One. 2013 Oct 11;8(10):e77643. doi: 10.1371/journal.pone.0077643 (PMC3795673; doi:10.1371/journal.pone.0077643)

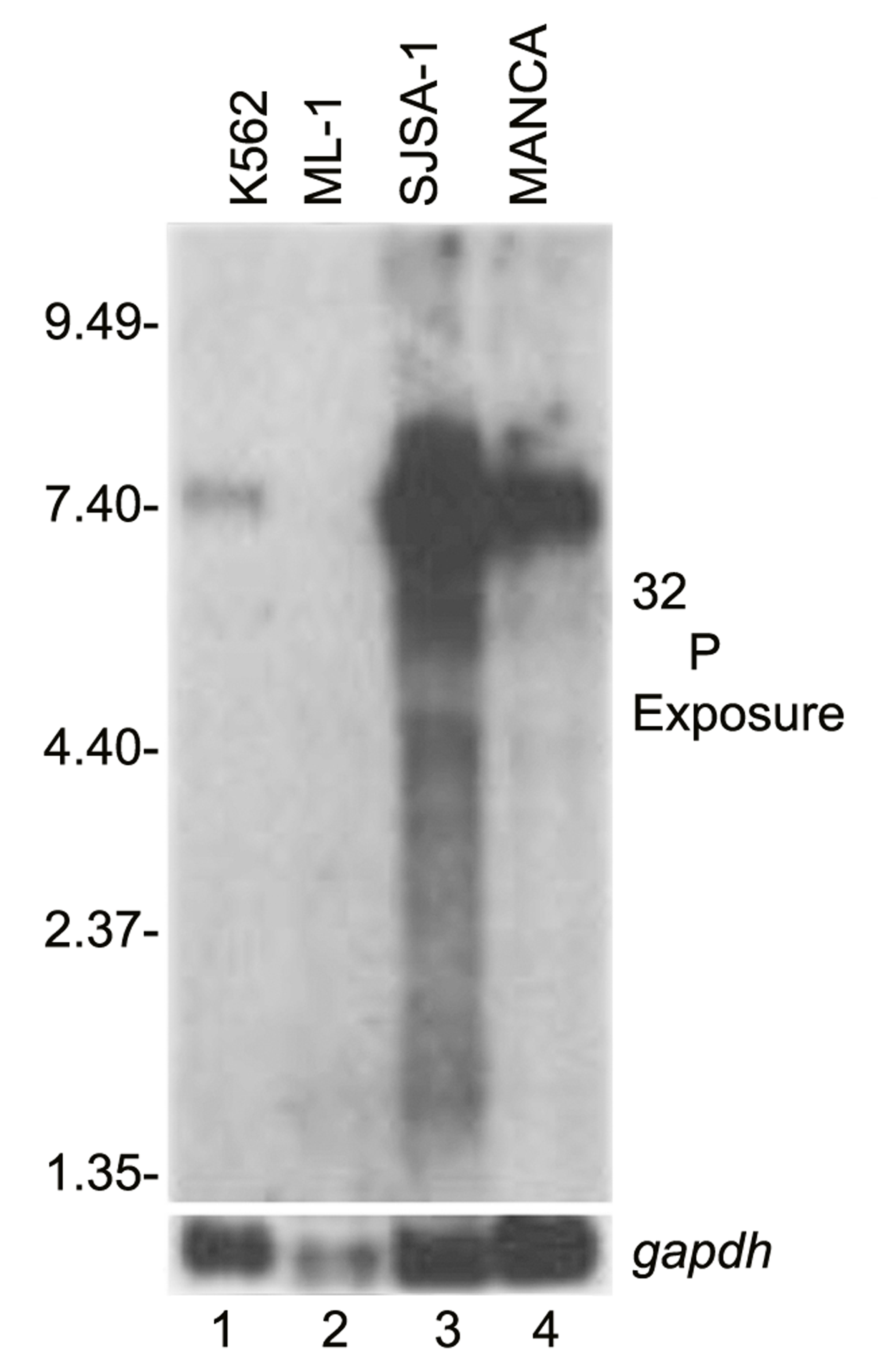

Supplement: Figure S1 — MDM2 over-expressing cells have high mdm2 transcript levels. Northern blot quantitation of total RNA from untreated K562, ML-1, SJSA-1 and MANCA, cells. RNA samples were electrophoresed onto a 1% denaturing formaldehyde agarose gel and transferred to a nylon membrane. The northern blot was probed with an exon 12-specific probe for mdm2 and exposed to film for transcript detection. α 32P dCTP was used as a radioactivity source for the probe labeling. GAPDH was used as a normalizer for RNA levels. Relative mdm2 message for lanes 1 - 4 was an average of 1, 2.2, 6 and 4 respectively shown in Figure 1. (TIF) [file pone.0077643.s001.tif]

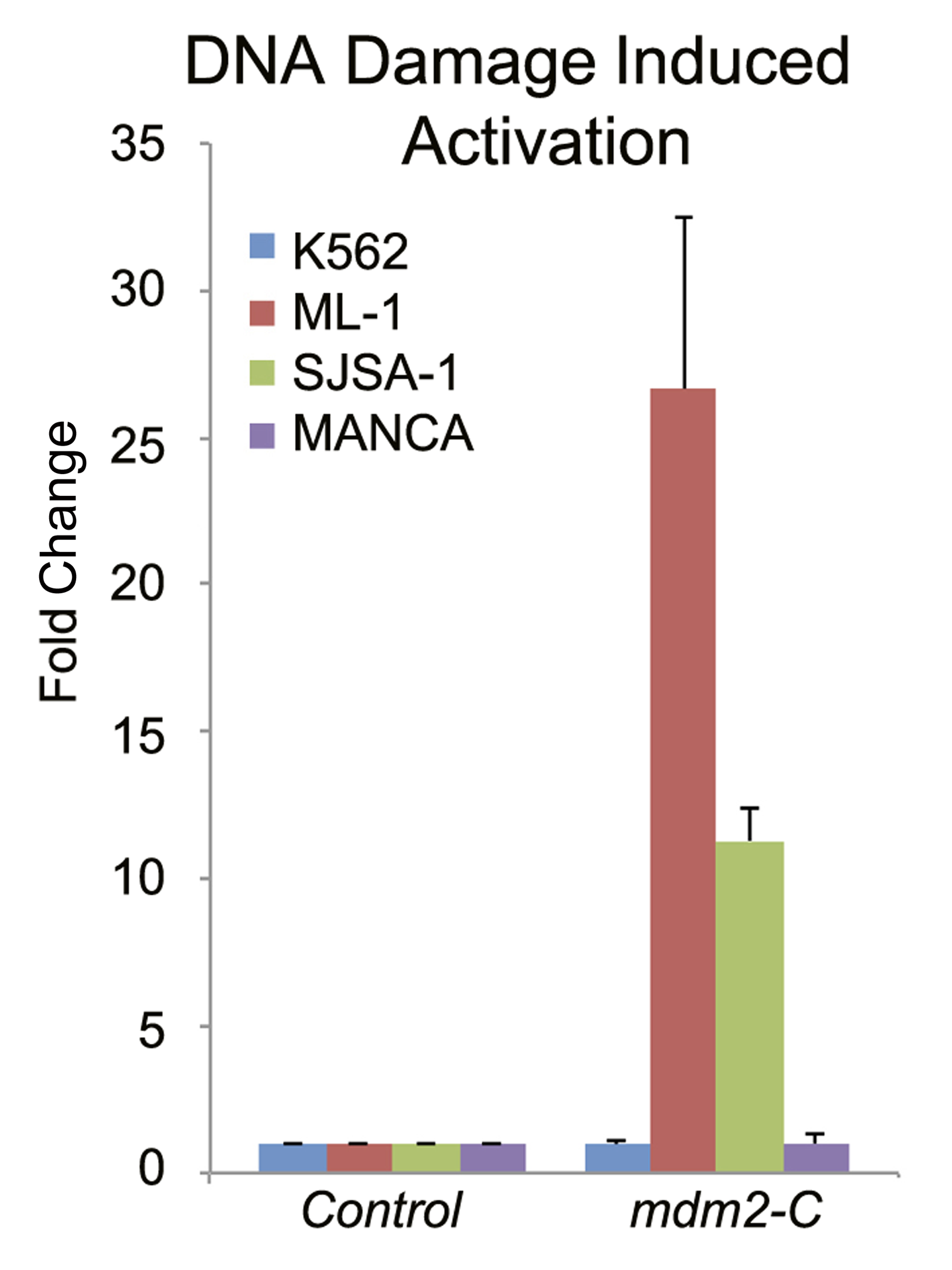

Supplement: Figure S2 — Etoposide activated transcription of mdm2 is compromised in MDM2 over-expressing cells. qRT-PCR of RNA from mdm2-C genes using syber green technology after induction of p53 via 8μM etoposide for 3 hours. Each cell line was normalized to its own control sample for fold activation and gapdh for RNA levels. An average of three independent experiments is shown. Error bars indicate standard error. (TIF) [file pone.0077643.s002.tif]

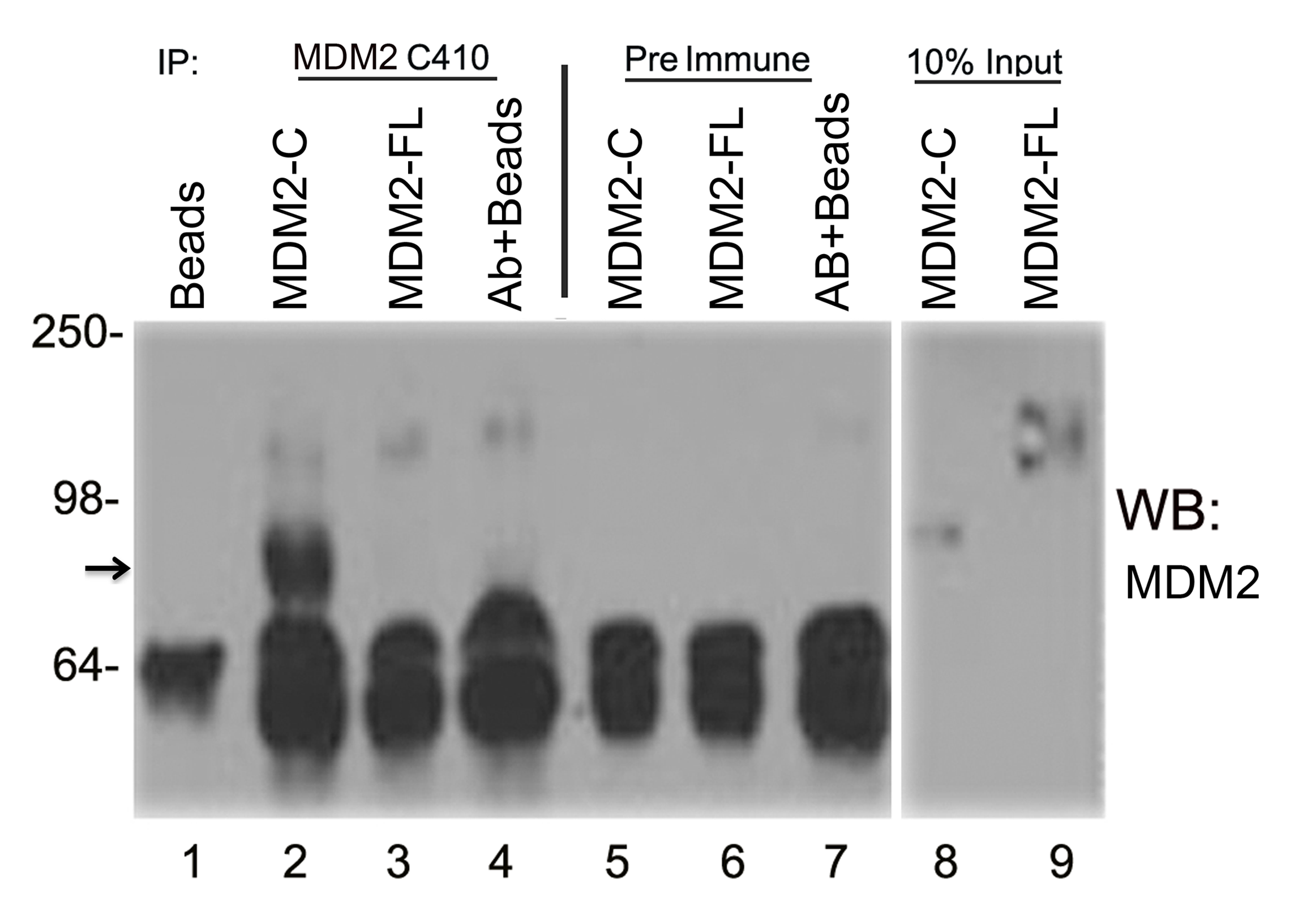

Supplement: Figure S3 — MDM2 C410 antibody is specific to MDM2-C protein. Immunoprecipitation of in vitro translated MDM2-FL and MDM2-C proteins using MDM2 C410 and pre-immune polyclonal serum antibodies. MDM2 monoclonal antibody mix (4B2, 2A9, 4B11) was utilized for protein identification. Wheat germ lysate without plasmid DNA was used as a negative control. HRP-conjugated anti-mouse and anti-rabbit were used as secondary antibodies. This is a representative of three independent experiments. Arrow represents MDM2-C band. (TIF) [file pone.0077643.s003.tif]

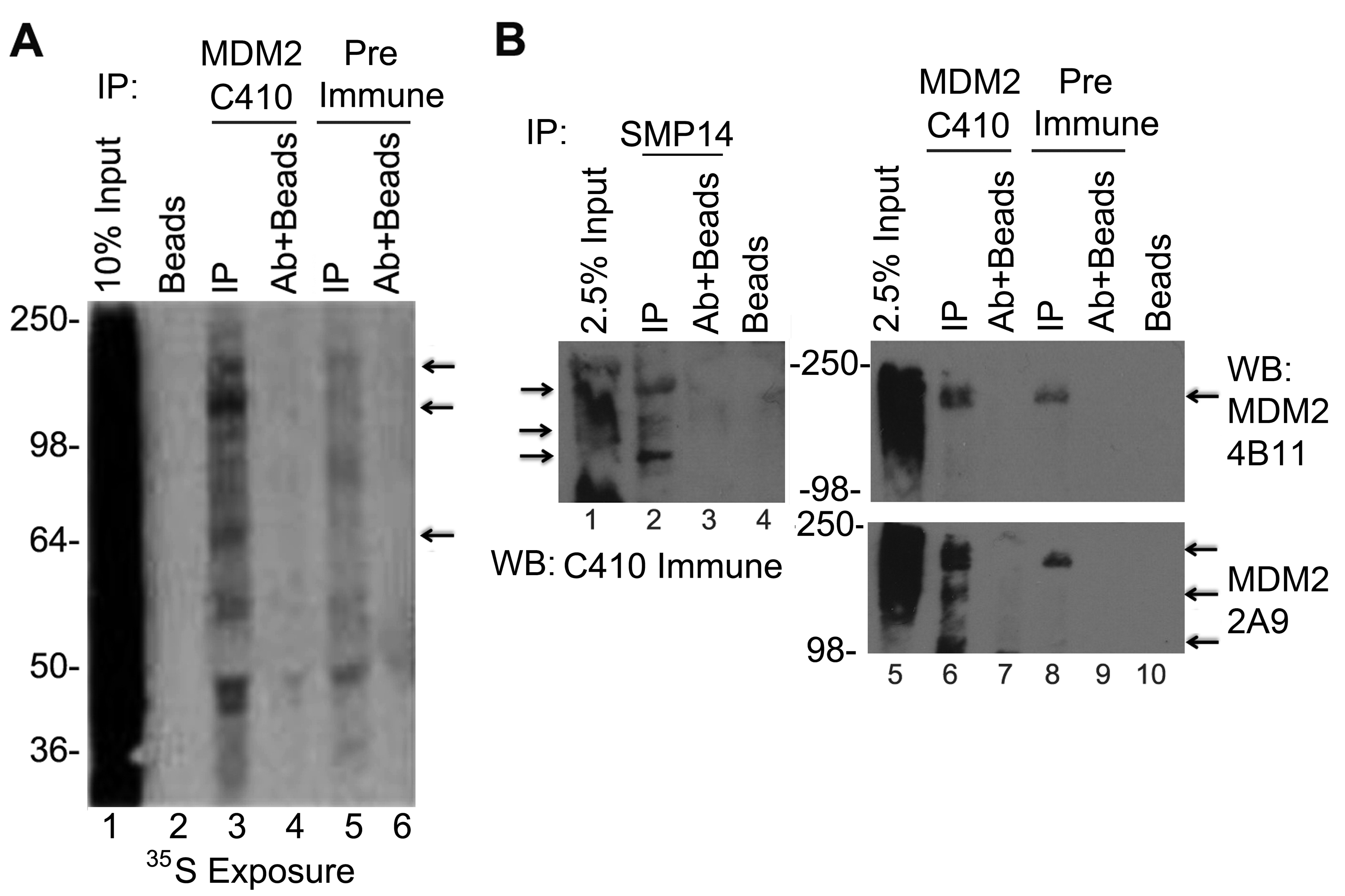

Supplement: Figure S4 — MDM2-C interacts with MDM2-FL and other cellular proteins invivo. A. Immunoprecipitation of 35S Methionine radioactive labeled MANCA whole cell extracts using Mdm2 C410 and pre-immune antibodies. Samples were electrophoresed on a 10% SDS-PAGE gel, transferred to nitrocellulose membrane and exposed to film for protein detection. Arrows represent MDM2 protein bands. B. Immunoprecipitation from MANCA whole cell extract using MDM2 antibody, SMP14. Samples were electrophoresed on a 10% SDS-PAGE gel, transferred to a nitrocellulose membrane and probed with MDM2 C410 polyclonal serum antibody. Immunoprecipitation was also performed with the MDM2 C410 and pre immune antibodies. Samples were electrophoresed on a 10% SDS-PAGE gel, transferred to a nitrocellulose membrane and probed with MDM2 4B11 monoclonal serum antibody. The membrane was sequentially re-probed with MDM2 2A9 monoclonal antibody. HRP-conjugated anti-mouse and anti-rabbit were used as secondary antibodies. Arrows represent MDM2-C interacting protein bands. (TIF) [file pone.0077643.s004.tif]
